# Mechanical sensing protein PIEZO1 controls osteoarthritis *via* glycolysis mediated mesenchymal stem cells-Th17 cells crosstalk

**DOI:** 10.1038/s41419-025-07577-1

**Published:** 2025-04-01

**Authors:** Yikun Zhou, Mingzhao Li, Shuai Lin, Zilu Zhu, Zimeng Zhuang, Shengjie Cui, Liujing Chen, Ran Zhang, Xuedong Wang, Bo Shen, Chider Chen, Ruili Yang

**Affiliations:** 1 Department of Orthodontics, Peking University School and Hospital of Stomatology, National Center for Stomatology & National Clinical Research Center for Oral Diseases & National Engineering Research Center of Oral Biomaterials and Digital Medical Devices, Beijing Key Laboratory of Digital Stomatology & NHC Key Laboratory of Digital Stomatology & NMPA Key Laboratory for Dental Materials Beijing, Beijing, China; 2 Department of Orthodontics, School and Hospital of Stomatology, Wuhan University, Wuhan, China; 3 Department of Oral Pathology, Peking University School and Hospital of Stomatology, Beijing, China; 4 National Institute of Biological Sciences, Beijing, China, Tsinghua Institute of Multidisciplinary Biomedical Research, Tsinghua University, Beijing, China; 5 Department of Oral and Maxillofacial Surgery and Pharmacology, University of Pennsylvania School of Dental Medicine, Philadelphia, PA USA

**Keywords:** Calcium and phosphate metabolic disorders, Chronic inflammation

## Abstract

Aberrant mechanical stimuli can cause tissue attrition and activate mechanosensitive intracellular signaling, impacting the progression of osteoarthritis (OA). However, the precise relationship between mechanical loading and bone metabolism remains unclear. Here, we present evidence that Piezo1 senses the mechanical stimuli to coordinate the crosstalk between mesenchymal stem cells (MSCs) and T helper 17 (Th17) cells, leading to the deterioration of bone and cartilage in osteoarthritis (OA). Mechanical loading impaired the property of MSCs by inhibiting their osteo-chondrogenic differentiation and promoting inflammatory signaling to enhance Th17 cells. Mechanistically, mechanical stimuli activated Piezo1, thereby facilitating Ca^2+^ influx which upregulated the activity of Hexokinase 2(HK2), the rate-limiting enzyme of glycolysis. The resultant increase in glycolytic activity enhanced communication between MSCs and T cells, thus promoting Th17 cell polarization in a macrophage migration inhibitory factor (MIF) dependent manner. Functionally, *Wnt1cre; Piezo1*^*fl/fl*^ mice reduced bone and cartilage erosion in the temporomandibular joint condyle following mechanical loading compared to control groups. Additionally, we observed activated Piezo1 and HK2-mediated glycolysis in patients with temporomandibular joint OA, thereby confirming the clinical relevance of our findings. Overall, our results provide insights into how Piezo1 in MSCs coordinates with mechano-inflammatory signaling to regulate bone metabolism.

**Figure:**
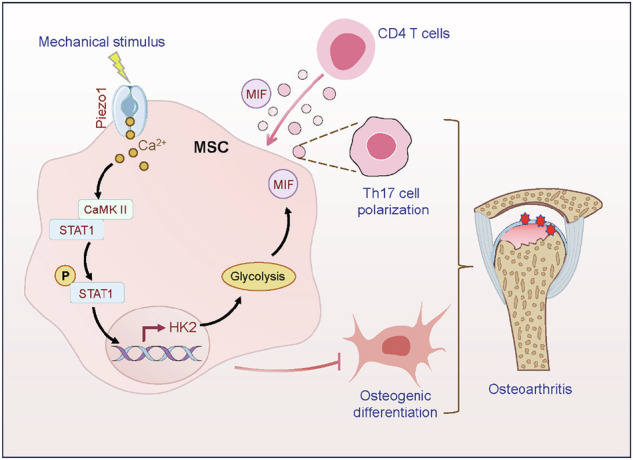
The schema shows that mechanical sensing protein PIEZO1 in MSCs controls osteoarthritis *via* glycolysis mediated MSCs and Th17 cells crosstalk in a MIF dependent manner.

The schema shows that mechanical sensing protein PIEZO1 in MSCs controls osteoarthritis *via* glycolysis mediated MSCs and Th17 cells crosstalk in a MIF dependent manner.

## Introduction

Bones are continuously subjected to mechanical loading during movement, and proper loading is crucial for maintaining bone homeostasis [1, 2]. However, excessive or chronic mechanical stimulus can lead to osteoarthritis (OA), a common disease that can cause severe pain and dysfunction in any joint, including the temporomandibular joint (TMJ) [3–5]. The pathology of the TMJOA is characterized by progressive cartilage degradation, subchondral bone remodeling, and chronic inflammation in synovial tissue [6, 7]. However, how the tissue and cells sense mechanical stimuli to initiate and activate the inflammatory response in degenerative diseases remains unknown.

Mesenchymal stem cells (MSCs) act as sensors of external forces and bear compression-loading [8–10]. We recently demonstrated that forces of varying magnitude can alter the protein profile of MSC-derived exosomes, thereby modulating bone metabolism during the orthodontic tooth movement process [11]. Mechanical unloading, on the other hand, leads to functional decline in MSCs and bone loss *via* dysregulation of TNF-α and mTOR signaling [12]. The Piezo1 channel has been reported to stimulate MSC migration by inducing ATP release to activate the MEK/ERK signaling pathway and thereby control osteoblast function [13, 14] However, whether the metabolism of MSCs affects their mechanical transduction potential and bone remodeling remains incompletely understood.

It has been reported that a non-infectious chronic inflammatory response, which is characterized by the infiltration of inflammatory cells into the synovial tissue or synovial fluid, is particularly apparent during the early stages of OA. This inflammatory process appears to contribute to both the degeneration of chondrocytes and bone attrition. Moreover, increased T cell infiltration has been observed in the synovial tissue during the early stages of OA [15], suggesting a role for the T cell-mediated inflammatory response in disease progression. During the immune response, T cells experience complex mechanical cues as they probe the surface of antigen-presenting cells [16]. T cells also generate internal forces *via* membrane tension and cytoskeletal reorganization [17–19]. Indeed, several studies have reported that the T cells receptor itself can act as a mechanosensor in response to tensile forces [17, 20–22]. Additionally, the mechanosensitive channel Piezo1 has been reported to restrain the regulatory T cells in autoimmune neuroinflammation [23]. However, whether mechanical loading affects T-cell function, and the immune response remains relatively unexplored.

In this study, we aimed to uncover the fine-tunable properties of mechanical stimuli in OA. We found the mechanical loading activated glycolysis *via* Piezo1 channels to drive crosstalk between MSCs and T cells in a manner dependent on macrophage migration inhibitory factor (MIF), which promoted the polarization of Th17 cells to drive OA development. These findings highlight the adaptive nature of the crosstalk between MSCs and inflammatory T cells in response to mechanical loadings by modulating their metabolic requirements, thus providing potential therapeutic targets for OA.

## Results

### Excessive mechanical stimulus leads to TMJOA

**Fig. 1:**
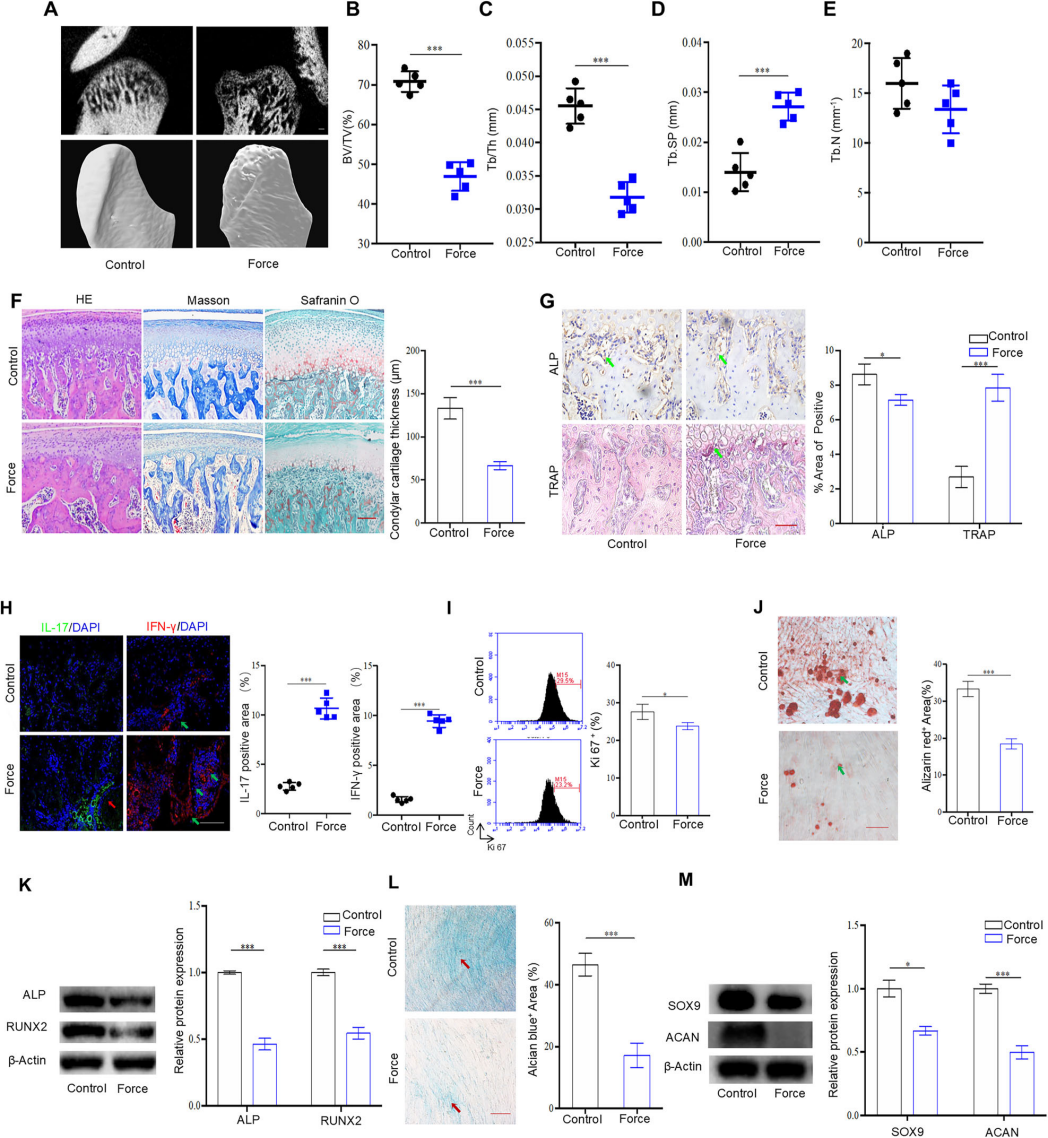
Excessive mechanical stimulus leads to temporomandibular joint OA. **A** The morphology of control and force-treated condyle tissues showed by micro-CT. Quantitative analysis of (**B**) BV/TV, (**C**) Tb.Th, (**D**) Tb.Sp and (**E**) Tb.N in subchondral bone of TMJ condyle determined by micro-CT analysis. **F** The morphology of control and force-treated condyle tissues showed by HE staining and Masson staining. **G** The expression of osteoblast and osteoclasts markers in control and force-treated TMJ condyles, assessed by ALP staining and TRAP staining. **H** The expression of IL-17 and IFN-γ in control and force-treated TMJ condyles. **I** The cell proliferation in control and force treated MSCs determined by flow cytometry. **J** Alizarin red S staining in control and force treated MSCs after osteogenesis induction for 21 days. **K** The expression levels of ALP and RUNX2 in control and force-treated MSCs analyzed by western blot. **L** Alcian blue staining in control and force-treated MSCs undergo chondrogenic differentiation for 21 days. **M** The expression levels of SOX9 and ACAN in control and force treated MSCs, as assessed by western blot. TMJ: temporomandibular joint, Scale bar: 100 μm (**F**, **H**, **J** and **L**); 50 μm (**G**); data are presented as the mean ± SEM, ^∗^*P* < 0.05; ^∗∗^*P* < 0.01; ^∗∗∗^*P* < 0.005.

To determine the effects of mechanical loading on bone metabolism, we established a TMJOA model by excessive mechanical stimulus using unilateral anterior crossbite (Fig. S1A). Microtomography examination of the control TMJ revealed a smooth and continuous surface of condyles, along with an even distribution of subchondral trabecular bone. In contrast, the mechanical loading group exhibited bone deterioration, erosion of the condylar surface, and discontinuity of the subchondral bone surface, accompanied by a decreased trabecular bone density (Fig. 1A–E). The thickness of the cartilage layer was remarkably reduced in the mechanical loading group, with an irregular chondrocyte arrangement, compared with the control group. Consistently, the loss of both the associated collagen and the cartilage matrix was confirmed by staining with Masson’s trichrome stain and safranin O (Fig. 1F). Notably, in the mechanical loading group, the number of alkaline phosphatase (ALP)-positive cells on the bone surface was reduced significantly, whereas that of tartrate-resistant acid phosphatase (TRAP)-positive osteoclasts in the subchondral bone was increased (Fig. 1G). Additionally, the infiltration of IL-17- and interferon-γ (IFN-γ)-positive inflammatory cells was greater in the mechanical loading group (Fig. 1H). These findings suggested that excessive mechanical loading contributes to TMJOA pathogenesis, including cartilage degradation, altered bone cell activity, and increased inflammatory cell infiltration.

MSCs have been reported to be sensitive to mechanical loading. Thus, we isolated MSCs from subchondral bone of the rat condyle and found that the MSCs expressed the mesenchymal stem cell markers CD90 and CD44, but not hematopoietic stem cell marker CD45, as assessed by flow cytometry (Fig. S1B). Next, we analyzed the properties of subchondral MSCs under mechanical stimulation revealing a decrease in MSC proliferation after mechanical loading (Fig. 1I). Moreover, the apoptosis of MSCs increased slightly after mechanical stimulation (Fig. S1C). The osteogenic differentiation of MSCs was impaired after mechanical loading, as evidenced by staining with Alizarin red S (Fig. 1J). The expression of osteogenic markers, including ALP and Runt-related transcription factor 2 (RUNX2), also decreased in MSCs subjected to mechanical force (Fig. 1K). Similarly, the chondrogenic differentiation capacity of MSCs was adversely affected by mechanical loading, as indicated by decreased Alcian blue staining (Fig. 1L). Correspondingly, the expression of chondrogenic markers, including sex-determining region Y-box 9 (SOX9) and aggrecan (ACAN), was decreased following mechanical loading (Fig. 1M). These findings indicated that abnormal mechanical loading adversely affects bone metabolism and compromises the osteochondrogenic differentiation potential of MSCs.

### The mechanosensory channel Piezo1 in MSCs drives TMJOA development

**Fig. 2:**
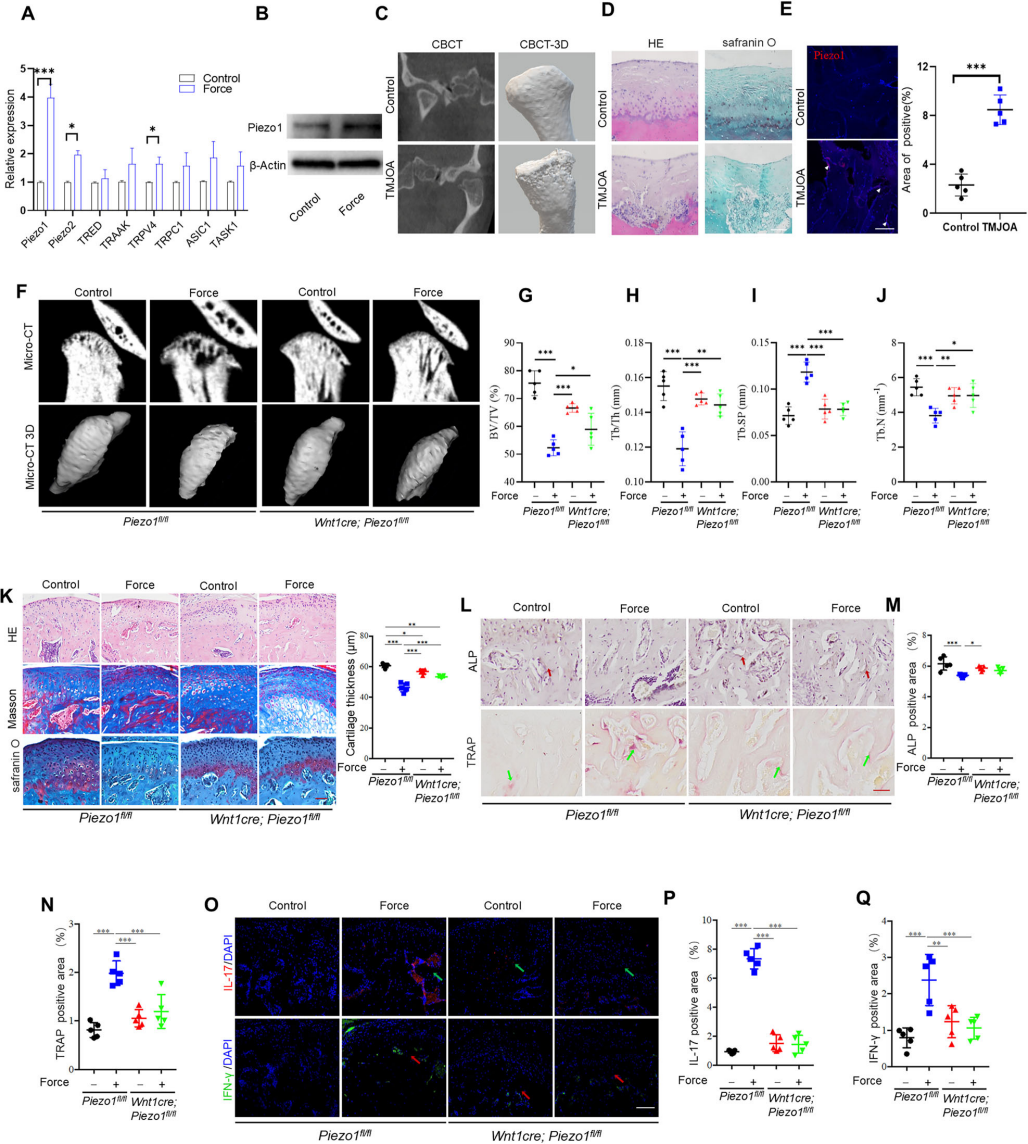
The mechanosensory channel Piezo1 in MSCs controls temporomandibular joint OA development. **A** The gene expression levels of mechanically activated ion channel in control and force treated groups determined by RT-PCR. **B** The MIF protein expression levels of Piezo1 in control and force treated MSCs. **C** The morphology of condyles in control and TMJOA human specimens were shown by CBCT analysis. **D** The histological characteristics of TMJ condyles in control and temporomandibular joint OA human specimens analyzed by HE staining and safranin O staining. **E** The expression of Piezo1 in control and TMJOA patients samples analyzed by immunofluorescence staining. **F** The morphology of TMJ condyle tissues in control and *Wnt1cre; Piezo1*^*fl/fl*^ mice with or without force treated groups was shown by micro-CT analysis. Quantitative analysis of (**G**) BV/TV, (**H**) Tb.Th, (**I**) Tb.Sp and (**J**) Tb.N in subchondral bone of TMJ condylar heads determined by micro-CT. **K** The histological characteristics of condyles in control and *Wnt1cre; Piezo1*^*fl/fl*^ mice with or without force treated groups was shown by HE staining, Masson staining and safranin O staining. **L**–**N** The ALP positive and TRAP-positive cells in control and *Wnt1cre; Piezo1*^*fl/fl*^ mice with or without force-treated groups were assessed by ALP staining and TRAP staining. **O**–**Q** The expression of IL-17 and IFN-γ in control and *Wnt1cre; Piezo1*^*fl/fl*^ mice with or without force treated condyle tissues. *TMJ* temporomandibular joint, Scale bar: 100 μm; data are presented as the mean ± SEM, ^∗^*P* < 0.05; ^∗∗^*P* < 0.01; ^∗∗∗^*P* < 0.005.

To determine how mechanical loading regulates bone metabolism, we analyzed the mechanosensitive channels, Piezo1and Piezo2, TWIK-related K^+^ channel (TREK-1), TRPC-1, ASIC1, TASK1 and TRAAK in MSCs. The results revealed that, among all these channels, Piezo1 expression increased most significantly after mechanical loading, as assessed by quantitative RT-PCR and western blotting (Fig. 2A, B). To determine the role of Piezo1 in MSC function, we knocked down Piezo1 *via* a specific short interfering RNA (SiRNA). The results showed that inhibition of Piezo1 by siRNA treatment partially restored the impaired osteochondrogenic differentiation of MSCs caused by mechanical loading, as evidenced by increased expression of ALP, RUNX2, SOX9, and aggrecan (Figure S2A–E). Strikingly, specimens from TMJOA patients showed a smooth and continuous surface of condyles in the control groups, whereas obvious condylar surface erosion and discontinuous bone surface were clearly detected in the arthritis group (Fig. 2C). Chondrocytes as well as the cartilage matrix were also decreased in the arthritis group (Fig. 2D). Moreover, the number of Peizo1 positive cells was increased more in the OA group than in the control group (Fig. 2E). These findings suggested that Piezo1 contributes to the sensing of mechanical stimuli and thus to the regulation of bone metabolism in OA.

We next generated *Wnt1cre; Piezo1*^*fl/fl*^ mice. These mice had slightly decreased bone density with no change in condyle morphology (Figs. 2F, S2F). We induced TMJOA in *Piezo1*^*fl/fl*^ and *Wnt1cre; Piezo1*^*fl/fl*^ mice and found that *Piezo1*^*fl/fl*^ mice had significant bone deterioration and cartilage degradation after mechanical loading, as assessed by microtomography and staining with hematoxylin and eosin, Masson’s trichrome stain, and safranin O; In contrast, *Wnt1cre; Piezo1*^*fl/fl*^ mice had almost no bone deterioration or cartilage degradation after mechanical loading (Fig. 2F–K). Consistently, mechanical force treatment decreased the ratio of ALP-positive cells in *Piezo1*^*fl/fl*^, but not in *Wnt1*^*Cre*^*; Piezo1*^*fl/fl*^ mice (Fig. 2L, M). Moreover, the presence of TRAP-positive cells was increased in *Piezo1*^*fl/fl*^, but not in *Wnt1*^*Cre*^*; Piezo1*^*fl/fl*^ mice after force loading (Fig. 2L-2N). Furthermore, the infiltration of IL-17 and IFN-γ-positive inflammatory cells were significantly increased in *Piezo1*^*fl/fl*^ mice after mechanical force loading, but this was not the case for *Wnt1*^*Cre*^*; Piezo1*^*fl/fl*^ mice (Fig. 2O–Q). These results implied that Piezo1 in MSCs could sense mechanical loading and regulate bone deterioration in the pathogenesis of TMJOA.

### Piezo1 in CD4^+^ T cells is dispensable for TMJOA progression

**Fig. 3:**
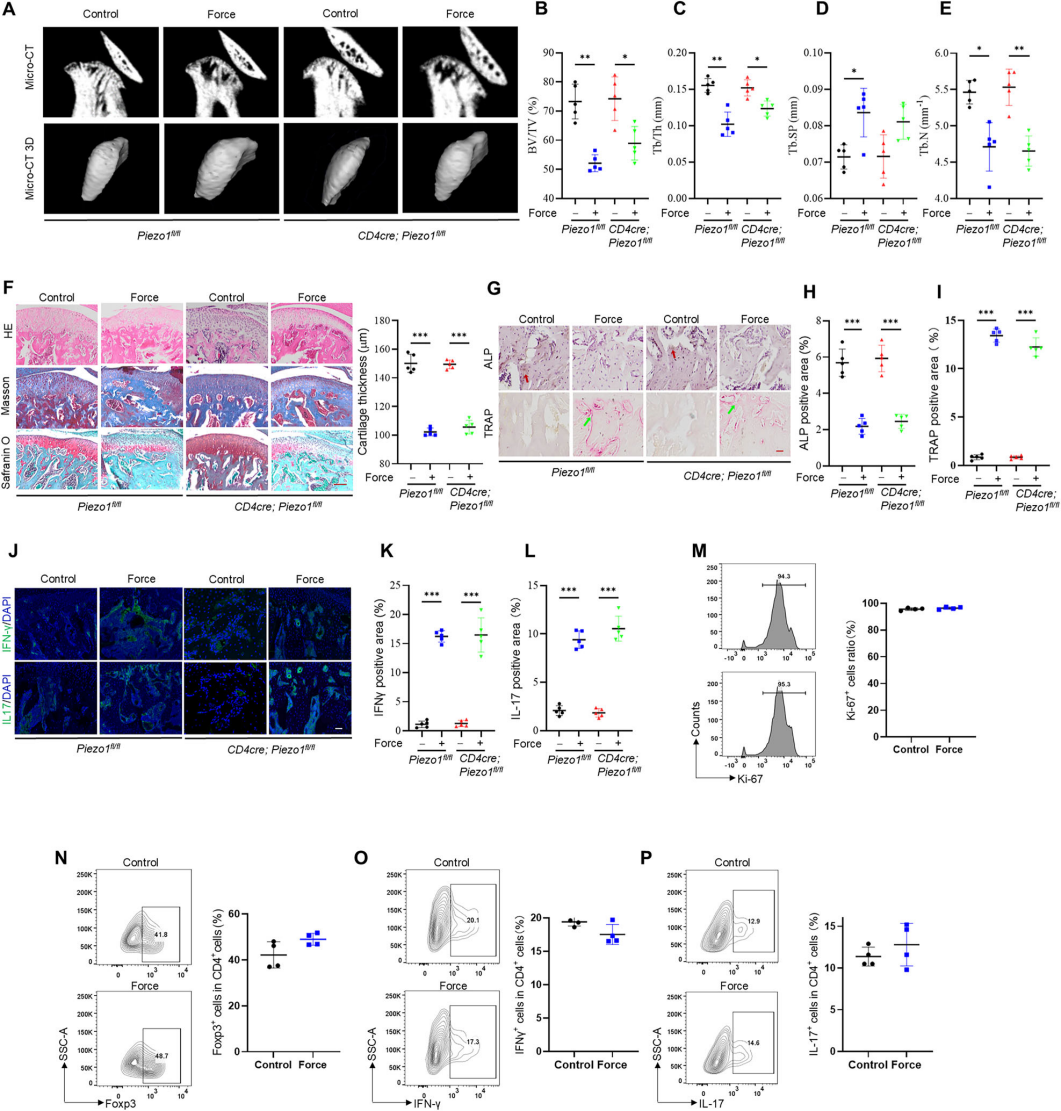
Piezo1 in CD4^+^ T cells failed to control temporomandibular joint OA development. **A** The morphology of TMJ condyle tissues in control, force treated and *CD4cre; Piezo1*^*fl/fl*^ mice and *CD4cre; Piezo1*^*fl/fl*^ mice with force treated groups was shown by micro-CT analysis. Quantitative analysis of (**B**) BV/TV, (**C**) Tb.Th, (**D**) Tb.Sp and (**E**) Tb.N in subchondral bone of TMJ condylar heads determined by micro-CT. **F** The histological characteristics of condyles in control, force treated and *CD4cre; Piezo1*^*fl/fl*^ mice and *CD4cre; Piezo1*^*fl/fl*^ mice with force treated groups were shown by HE staining, Masson staining, and safranin O staining. **G**–**I** The ALP positive and TRAP-positive cells in control, force treated and *CD4cre; Piezo1*^*fl/fl*^ mice and *CD4cre; Piezo1*^*fl/fl*^ mice with force treated groups were assessed by ALP staining and TRAP staining. **J**–**L** The expression of IL-17 and IFN-γin control, force treated and *CD4cre; Piezo1*^*fl/fl*^ mice and *CD4cre; Piezo1*^*fl/fl*^ mice with force treated groups condyle tissues. **M** The Ki-67 positive cells ratio in native CD4^+^ T cells from control and force treated groups. **N** Foxp3 positive cells ratio in native CD4^+^ T cells from control and force treated groups. **O** IFN-γ positive cells ratio in native CD4^+^ T cells from control and force treated groups. **P** IL-17 positive cells ratio in native CD4^+^ T cells from control and force treated groups. *TMJ* temporomandibular joint, Scale bar: 100 μm; data are presented as the mean ± SEM, ^∗^*P* < 0.05; ^∗∗^*P* < 0.01; ^∗∗∗^*P* < 0.005.

It has been reported that CD4^+^ T cells are present at the early stage of OA [15]. Next, we analyzed whether Piezo1 in CD4^+^ T cells contribute to TMJOA progression. First, we generated *CD4cre; Piezo1*^*fl/fl*^ mice and found that neither condyle morphology nor bone density differed significantly from *Piezo1*^*fl/fl*^ mice. Next, we induced TMJOA, which resulted in significant bone erosion and cartilage degeneration of the TMJ condyle in both *Piezo1*^*fl/fl*^ and *CD4cre; Piezo1*^*fl/fl*^ mice after mechanical stimulation (Figs. 3A–F, S3A). In addition, mechanical force treatment similarly decreased the ratio of ALP-positive cells and increased the presence of TRAP-positive cells both in *Piezo1*^*fl/fl*^ and *CD4cre; Piezo1*^*fl/fl*^ mice (Fig. 3G–I). Furthermore, mechanical stimulation increased the infiltration of IL-17 and IFN-γ-positive inflammatory cells both in *Piezo1*^*fl/fl*^ and *CD4cre; Piezo1*^*fl/fl*^ mice (Fig. 3J–L). These results indicated that Piezo1 in CD4^+^ T cells did not regulate TMJOA development directly.

Consistently, there was no significant difference in T helper cells ratio including Th1, Th17 and regulatory T (Treg) cells between control and *CD4cre; Piezo1*^*fl/fl*^ mice (Fig. S3B–D). Next, we isolated CD4^+^ T cells to analyze whether Piezo1 regulates T cell proliferation. Proliferation in vitro was found to be similar for naïve CD4^+^ T cells from control and *CD4cre; Piezo1*^*fl/fl*^ mice, as assessed by the abundance of Ki-67 positive cells (Fig. S3E). We next determined the effects of Piezo1 on the polarization of helper T cells. Naïve (CD4^+^CD44^low^CD62L^high^CD25^-^) T cells were stimulated in the presence of polarizing cytokines to induce Th1, Th17, and Treg cells in vitro. The results revealed that *CD4cre; Piezo1*^*fl/fl*^ differentiated into Th1 and Th17 inflammatory subsets to the same extent as control T cells, assessed by the expression of the signature inflammatory markers interferon-γ (IFN-γ) and interleukin-17 (IL-17), respectively (Fig. S3F,G). Moreover, the two groups showed the same percentage of fork head transcription factor Foxp3 expressing (Foxp3^+^) Treg cells in the presence of transforming growth factor–β (TGFβ) and IL-2 (Fig. S3H). Strikingly, mechanical stimulus treatment failed to alter the proliferation and polarization of T cells in vitro (Fig. 3M–P). Together, these results indicated that Piezo1 and a mechanical stimulus does not play a direct role in either CD4^+^ T cell proliferation or T cellpolarization.

### Mechanical stimulus promotes crosstalk between MSCs and T cells *via* MIF

**Fig. 4:**
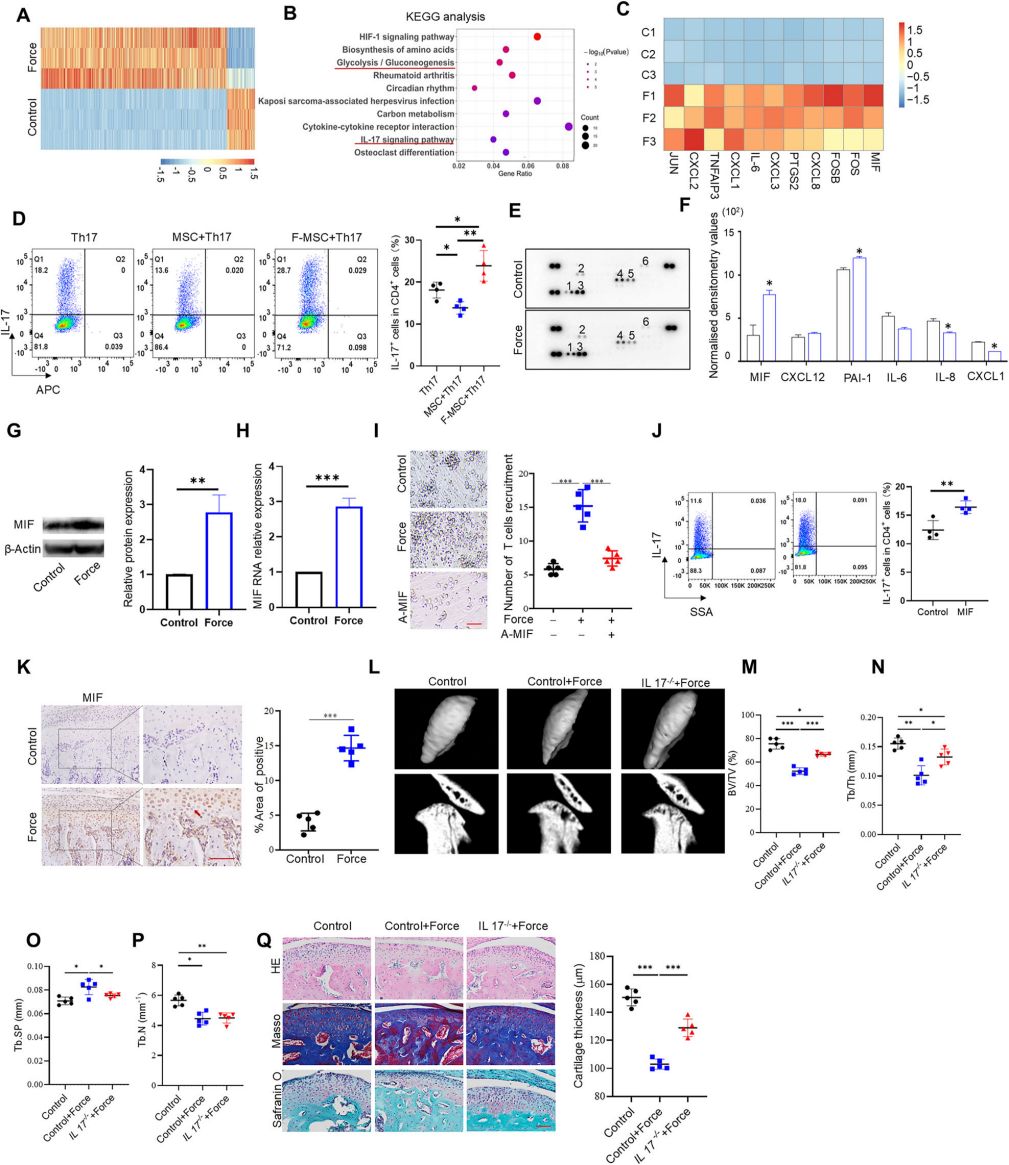
Mechanical stimulus promoted the crosstalk between MSCs with T cells *via* MIF. **A** A heatmap depicting the upregulated and downregulated genes between control and force-treated MSCs. **B** The KEGG analysis showed that IL-17 signaling pathway was one of the top ten different enriched clusters between control and force-treated MSCs. **C** A heatmap depicting the different gene expression related with IL-17 signaling pathway. **D** Th17 cell differentiation in control, co-cultured with MSCs, and co-cultured with mechanical stimulus pretreated MSCs groups was analyzed by flow cytometry. **E** Cytokine secretion profiles of MSCs from control and force-treated groups. **F** Semi-quantification of cytokine array results. **G** The protein expression levels of MIF in control and force-treated groups. **H** The gene expression levels of MIF in control and force-treated groups. **I** The recruitment of T cells by MSCs in control, force with or without Anti-MIF groups. **J** Th17 cell differentiation in control and MIF-treated groups was analyzed by flow cytometry. **K** The expression of MIF in control and force-treated condyle tissues, was assessed by immunohistochemistry staining. **L** The morphology of TMJ condyle tissues in control, force-treated and *IL-17*^*-/-*^ mice with force-treated groups was shown by micro-CT analysis. Quantitative analysis of (**M**) BV/TV, (**N**) Tb.Th, (**O**) Tb.Sp and (**P**) Tb.N in subchondral bone of TMJ condylar heads determined by micro-CT. **Q** The histological characteristics of condyles in control, force-treated, and *IL-17*^*-/-*^ mice with force-treated groups were shown by HE staining, Masson staining, and safranin O staining. *TMJ* temporomandibular joint, Scale bar: 100 μm; data are presented as the mean ± SEM, ^∗^*P* < 0.05; ^∗∗^*P* < 0.01; ^∗∗∗^*P* < 0.005.

The interaction between MSCs and immune cells plays a crucial role in the inflammatory response [24]. Next, we performed RNA sequencing (RNA-seq) of MSCs that had been subjected to force treatment. Figure 4A presents a heatmap analysis depicting the significantly upregulated and downregulated genes (fold change > 2, *p* < 0.05). Notably, IL-17 pathways emerged as significantly enriched signaling clusters after mechanical force treatment, as determined by Kyoto Encyclopedia of Genes and Genomes annotation (KEGG) and gene set enrichment analysis (GSEA) (Fig. 4B, C). MSCs were then co-cultured with T cells in the presence of Th17 polarization cytokines, the MSCs could slightly inhibit Th17 cell differentiation, whereas MSCs first exposed to a mechanical stimulus could enhance Th17 cell polarization compared with the no stimulated MSC control group (Fig. 4D). MSCs can secrete a variety of cytokines, such as M-CSF and MCP-1, to recruit monocytes and immune cells [25–27]. We therefore used cytokine array analysis to identify molecules that contribute to Th17 polarization induced by MSCs under mechanical stimulation. The analysis revealed that the change of migration inhibitor factor (MIF) was the most significant response to mechanical stimuli (Fig. 4E, F). The increased expression of MIF was confirmed by western blotting and quantitative RT-PCR (Fig. 4G, H). MIF has been reported to support the homing of monocytes to peripheral osteolytic lesions [27]. Subsequently, we investigated whether a mechanical stimulus could regulate T cell recruitment. The results demonstrated that MSCs could effectively recruit T cells, and this process could be blocked by a MIF neutralizing antibody (Fig. 4I). Moreover, MIF could enhance the polarization of Th17 cells (Fig. 4J). Consistently, the level of MIF was significantly increased in the joint of the mechanical stress-induced OA group compared with the control group (Fig. 4K). To verify the role of MIF, we utilized the MIF-neutralizing antibody to treat TMJOA. Indeed, the antibody could limit bone deterioration in the condyle compared with the TMJOA group (Fig. S4A–E). Additionally, cartilage thickness and chondrocyte arrangement were remarkably improved after antibody treatment (Fig. S4F, G). The MIF-neutralizing antibody also effectively inhibited the force-activated TRAP-positive osteoclasts and promoted the activity of ALP-positive osteoblasts (Fig. S4H). Furthermore, the increased infiltration of IL-17 and IFN-γ-positive inflammatory cells in TMJOA was markedly reduced upon treatment with the MIF-neutralizing antibody (Fig. S4I). These findings highlighted MIF is one of the mediators that manipulate bone metabolism under mechanical micro-environment.

To verify the role of Th17 cells in mechanical stress-induced TMJOA, we induced TMJOA in IL-17^-/-^ mice, these IL-17^-/-^ mice had lesser bone resorption and cartilage erosion in TMJOA after mechanical loading compared with the control ones (Fig. 4L–Q). Collectively, these results indicated that MIF could mediate crosstalk between MSCs and Th17 cells to control bone homeostasis under mechanical stimulation.

### Mechanical stimulus promotes MIF secretion *via* hexokinase 2 (HK2)- mediated glycolysis

**Fig. 5:**
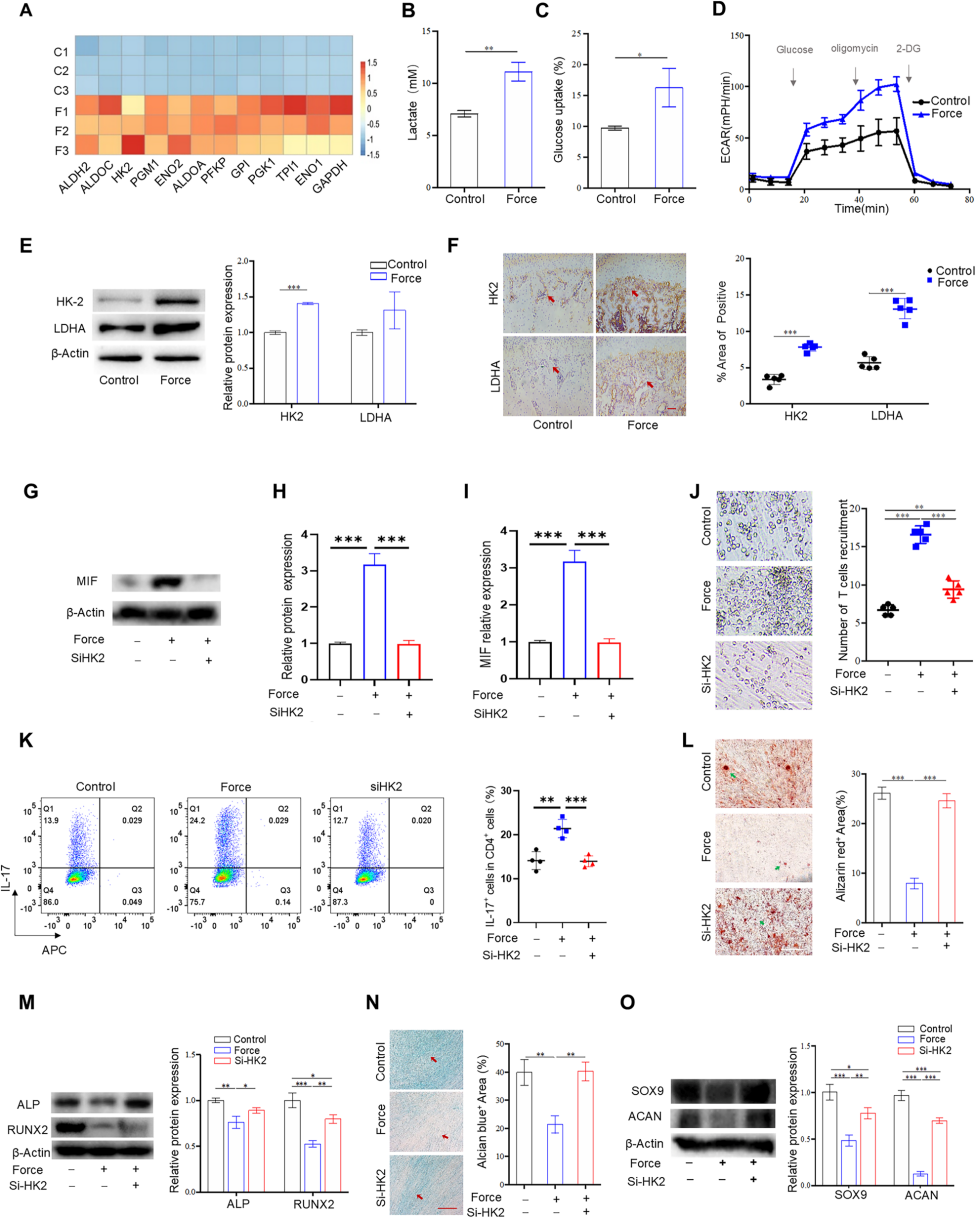
Mechanical stimulus promoted MIF secretion *via* HK2 mediated glycolysis. **A** A heatmap depicting the different gene expressions related with the glycolysis pathway. **B** The lactate level produced by control and force-treated MSCs. (C)The glucose consumption rate in control and mechanical force treated MSCs. **D** Extracellular acidification rate (ECAR) in control and force-treated MSCs. **E** The expression of glycolytic enzymes HK2 and LDHA in control and force-treated MSCs, assessed by western blot. **F** The expression of HK2 and LDHA in control and force-treated TMJ condyle, was assessed by immunohistochemistry staining. **G** The protein expression levels of MIF in control, force with or without SiHK2 pre-treated MSCs groups. **H** Semi-quantification of the protein expression levels of MIF. **I** The gene expression levels of MIF in control, force with or without SiHK2 pre-treated MSCs groups. **J** The recruitment of T cells by MSCs in control, force with or without SiHK2 groups. **K** Th17 cell differentiation in control, force with or without SiHK2 groups was analyzed by flow cytometry. **L** The osteogenic differentiation of MSCs in control, force with or without SiHK2 pre-treated groups after osteogenic induction for 21 days, assessed by Alizarin red S staining. **M** The protein expression levels of ALP and RUNX2 in control, force with or without SiHK2 pre-treated groups. **N** The chondrogenic differentiation of MSCs in in control, force with or without SiHK2 pre-treated groups, as assessed by Alcian blue staining. **O** The protein expression levels of SOX9 and ACAN in control, force with or without SiHK2 pre-treated groups. *TMJ* temporomandibular joint, Scale bar: 100 μm; data are presented as the mean ± SEM, ^∗^*P* < 0.05; ^∗∗^*P* < 0.01; ^∗∗∗^*P* < 0.005.

To investigate the mechanisms by which a mechanical stimulus could alter the secretion of MIF from MSCs. RNA-sequencing data for control and force-treated MSCs were further analyzed. Notably, glycolytic pathways emerged as one of the most significantly enriched signaling clusters after mechanical force treatment, as determined by KEGG) and GSEA (Figs. 4B, 5A, and S5A). Subsequently, we found that mechanical stimuli markedly increased lactate production and glucose uptake by MSCs (Fig. 5B and C). To further determine the impact of mechanical stimulation on fuel utilization and glycolytic flux, we assessed the extracellular acidification rate (ECAR), which is the main indicator of glycolysis. The extracellular acidification rate was significantly increased after mechanical stimulation (Fig. 5D). To confirm the involvement of mechanical loading in glycolysis regulation, we analyzed the expression of key catalytic enzymes in the glycolysis pathway, including hexokinases 1 and 2 (HK1 and HK2), the M2 isoform of pyruvate kinase (PKM2), 6-phosphofrocto-2-kinase and lactate dehydrogenase A (LDHA). The results showed that HK2 and LDHA mRNAs were remarkably increased after mechanical force stimulation (Fig. S5B). The upregulation of HK2 and LDHA was further validated by western blotting (Fig. 5E). Moreover, we observed a significant increase in the expression of HK2 and LDHA in the subchondral bone of the TMJ condyle of the OA group (Fig. 5F). Next, we analyzed whether glycolysis could regulate the crosstalk between MSCs and T cells and found that siRNA-mediated knockdown of HK2 could block mechanical force induced MIF expression (Figs. 5G–I, S5C,D). Moreover, the recruitment of T cells induced by MSCs that had been subjected to mechanical force was also inhibited by the HK2-specific siRNA (Fig. 5J). Consistently, Th17 polarization was also inhibited by this siRNA (Fig. 5K). Furthermore, the impaired osteochondrogenic differentiation of MSCs caused by mechanical force was partially restored by the HK2 siRNA (Fig. 5L), and this restoration was accompanied by increased expression of the osteochondrogenic differentiation molecules, including ALP, RUNX2, SOX9 and ACAN (Fig. 5L–O). These findings suggested that mechanical stimulus could promote HK2-mediated glycolysis to control crosstalk between MSCs and Th17 cells *via* MIF.

### Mechanical stimulus activates glycolysis *via* Piezo1-STAT1 signaling

**Fig. 6:**
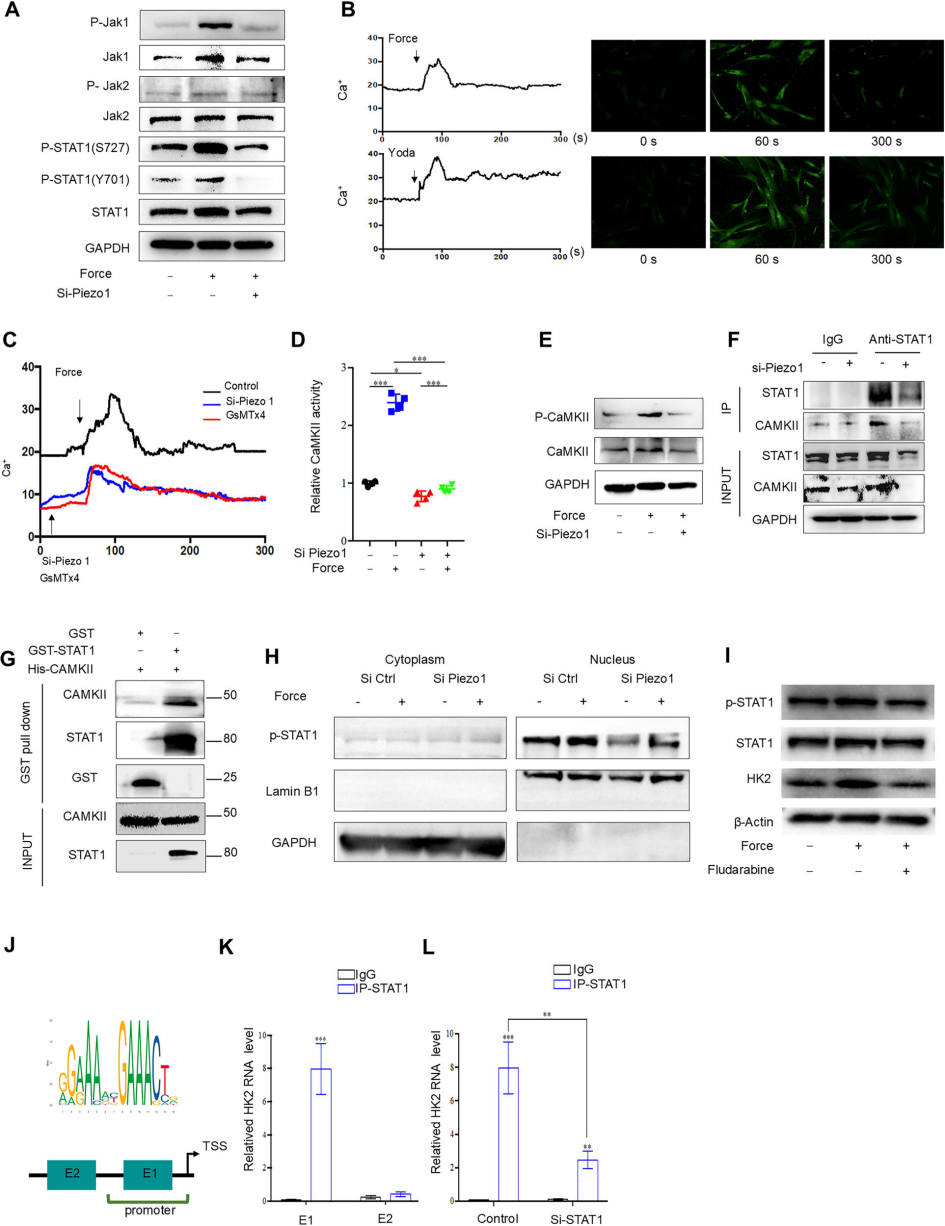
Mechanical stimulus activated glycolysis *via* STAT1 signaling. **A** The protein expression levels of p-JAK1/JAK1, p-JAK2/JAK2, and p-STAT1/STAT1 in control, the force with or without Piezo1 siRNA treated MSCs. **B** The Ca^2+^ influx induced by force and Yoda treatment in MSCs. **C** The Ca^2+^ influx induced by force treatment in MSCs was inhibited with Piezo1 inhibitor GsMTx4 or Piezo1 siRNA treatment. **D**, **E** The enzymatic activity of CaMKII and expression of phosphorylated CaMKII in control, force with or without Piezo1 siRNA treated for 24 h MSCs. **F** Co-immunoprecipitation assay showing that CaMKII interact STAT1 directly, which was attenuated by Piezo1 siRNA treatment. **G** GST pull-down assays were performed with GST-fusion proteins containing the portions of CaMKII and in vitro translated STAT1. The bound proteins were separated by SDS/PAGE and visualized by autoradiography. **H** The localization of p-STAT1 in the nuclei and cytoplasm fraction in control, force with or without Piezo1 siRNA treated MSCs. **I** The expression of p-STAT1 and HK2 in control, the force with or without fludarabine pre-treated MSCs analyzed by western blot. **J** It’s predicted that STAT1 could target on the promoter region of the HK2 gene hinted by JASPAR database. **K**, **L** The enrichment of STAT1 on the element 1 and element 2 of HK2 promoter was determined by ChIP-qPCR. Scale bar: 100 μm; data are presented as the mean ± SEM, ^∗^*P* < 0.05; ^∗∗^*P* < 0.01; ^∗∗∗^*P* < 0.005.

We proceeded to analyze changes in certain metabolic pathways following mechanical loading in MSCs using RNA-sequencing data. The results implicated the involvement of the JAK/STAT pathway in the interplay between glycolysis and bone metabolism (Fig. S6A,B). Notably, the kinase activity of the JAK/STAT pathway results in the phosphorylation of several downstream effectors involved in processes such as hematopoiesis, immune fitness, tissue repair, and adipogenesis [28]. Specifically, our analysis of the STAT1 pathway revealed that mechanical stimulation led to the activation of JAK2, but not JAK1, leading to the phosphorylation of STAT1 at Ser727 rather than Y701. Importantly, the increase in phosphorylated STAT1 induced by mechanical force was inhibited after treatment of MSCs with a Piezo1-specific siRNA (Fig. 6A). To investigate how mechanical force could activate STAT1 signaling, we assessed calcium influx using Yoda, a selective agonist of Piezo1, or mechanical loading treatment. The results showed that both Yoda and a mechanical stimulus elicited Ca^2+^ flux, in line with previous reports [29–32] (Fig. 6B). Furthermore, the Ca^2+^ influx induced by force treatment in MSCs was inhibited by either the Piezo1 inhibitor GsMTx4 or Piezo1 siRNA (Fig. 6C). One of the main consequences of increased Ca^2+^ influx is the phosphorylation of the Ca^2+^calmodulin-dependent kinase II (CaMKII), a family of serine/threonine kinases involved in various cellular process [33]. Indeed, the mechanical force could stimulate CaMKII activity and phosphorylation, which was blocked by the Piezo1 siRNA (Fig. 6D-6E). Additionally, GST pull-down assay revealed an interaction between STAT1 and CaMKII (Fig. 6F). Furthermore, co-immunoprecipitation of whole cell extracts of MSCs demonstrated a direct interaction between endogenous STAT1 and CaMKII, and this also was inhibited by the Piezo1 siRNA (Fig. 6G). Consistently, cell fractionation analysis showed that phosphorylated-STAT1 activated by mechanical force, was predominantly located in the nucleus, and this localization was partially hindered by the Piezo1 siRNA (Fig. 6H). These results indicated that the mechanical loading elicits a calcium influx *via* the Piezo1 channel to induce the interaction between the calcium-dependent CamKII and STAT1, thus leading to the phosphorylation of STAT1.

To understand how STAT1 signaling regulates glycolysis in MSCs, we analyzed the interaction between STAT1 and HK2. We used fludarabine, an inhibitor of STAT phosphorylation, to treat MSCs and found that HK2 expression was decreased compared with the mechanical loading group (Fig. 6I). These results indicated that STAT1 plays an important role in the upregulation of HK2 in response to a mechanical stimulus. Next, we utilized the JASPAR database (http://jaspar.genereg.net/) to screen the interaction for the putative binding sites for STAT1 within the HK2 promoter (Fig. 6J). A chromatin immunoprecipitation assay was then performed to verify this interaction. The results demonstrated that STAT1 could directly bind to element 1, but not element 2, within the HK2 promoter (Fig. 6K). Furthermore, the binding of STAT1 to the promoter was partially attenuated upon treatment with a STAT1-specific siRNA (Fig. 6L). Functionally, treatment of MSCs with the STAT1 inhibitor fludarabine restored the osteochondrogenic differentiation of MSCs inhibited by mechanical loading and increased the expression of ALP, RUNX2, SOX9, and ACAN (Fig. S6C–F). These findings implied that mechanical stimuli could activate glycolysis *via* Piezo1-STAT1 signaling, which directly targeted the HK2 promoter to control MIF release.

### TMJOA can be alleviated by inhibiting glycolysis

**Fig. 7:**
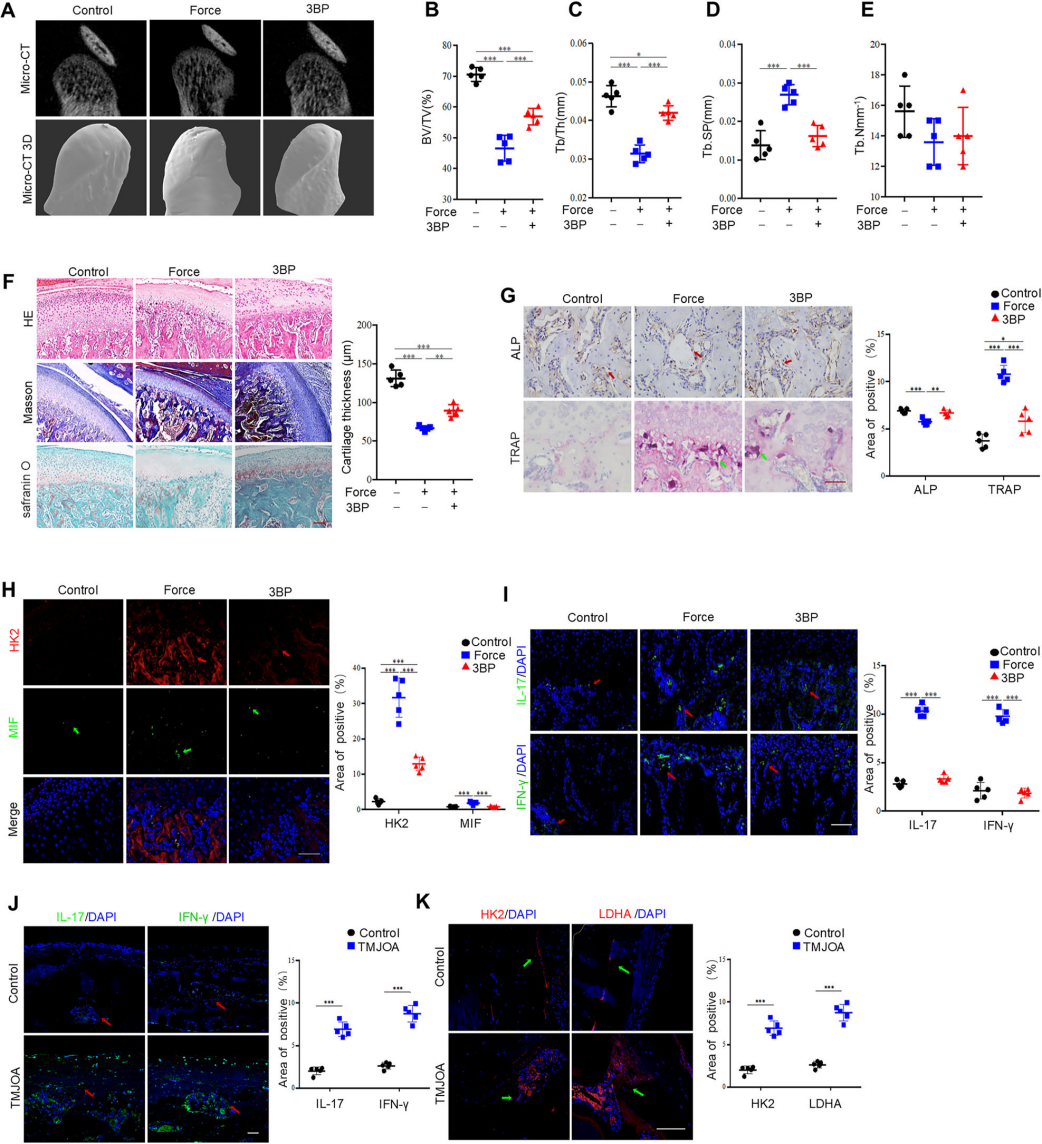
Temporomandibular joint OA could be alleviated by inhibiting glycolysis. **A** The morphology of TMJ joint in control, force with or without 3-BP treated groups was shown by micro-CT analysis. Quantitative analysis of (**B**) BV/TV, (**C**) Tb.Th, (**D**) Tb.Sp and (**E**) Tb.N in subchondral bone of temporomandibular joint determined by micro-CT measurements. **F** The histological characteristics of TMJ subcondral bone in control, force with or without 3-BP treated groups, showed by HE staining, Masson staining and safranin O staining. **G** The ALP and TRAP-positive cells in control, force with or without 3-BP treated TMJ subchondral bone were shown by ALP staining and TRAP staining. The expression of HK2, MIF (**H**) and IL-17, IFN-γ (**I**) in control, force with or without 3-BP treated groups, assessed by immunofluorescence staining. **J** The expression of IL-17 and IFN-γ in control and TMJOA human condyle specimens, assessed by immunofluorescence staining. **K** The expression of HK2 and LDHA in control and TMJOA human condyle specimens, was assessed by immunofluorescence staining. *TMJ* temporomandibular joint, Scale bar: 100 μm; data are presented as the mean ± SEM, ^∗^*P* < 0.05; ^∗∗^*P* < 0.01; ^∗∗∗^*P* < 0.005.

To investigate the potential impact of force-induced glycolysis on TMJOA, we injected the glycolysis inhibitor 3-BP into the animal model. The application of 3-BP effectively attenuated bone erosion on the condylar surface and bone resorption in the subchondral bone induced by mechanical loading (Fig. 7A–E). Moreover, the group treated with 3-BP had increased cartilage thickness with slightly irregular chondrocyte arrangement, and staining of the cartilage matrix was similar to that of the arthritis group (Fig. 7F). The decreased number of ALP-positive cells on the bone surface in the mechanical loading group was partially restored by 3-BP treatment. Furthermore, the inhibition of glycolysis by 3-BP during mechanical stimulation successfully inhibited the infiltration of force-activated TRAP-positive osteoclasts (Fig. 7G). These results indicated that mechanical loading disrupts bone remodeling *via* the glycolytic pathway mediated by HK2.

Interestingly, the MIF positive cells were considerably increased in the TMJOA group compared with the control group, and this increase was mitigated by the inhibition of glycolysis (Fig. 7H). Similarly, the number of inflammatory cells positive for IL-17 and IFN-γ was greater in the arthritis group, which was also reduced after 3-BP injection (Fig. 7I). Moreover, the infiltration of IL-17- and IFN-γ-positive cells was greater in the specimens from TMJOA patients compared with the control one (Fig. 7J). The proportions of HK2-positive cells and LDHA-positive cells in the bone destruction area were remarkably higher in TMJOA patients compared with the control ones (Fig. 7K). These results indicated that mechanical loading disrupts bone remodeling *via* Piezo1/HK2-mediated glycolysis.

Taken together, these results emphasize that the mechanical sensor Piezo1 channel drives crosstalk between MSCs and T cells in an MIF-dependent manner to initiate TMJOA, suggesting potential therapeutic targets for OA prevention and treatment (Fig. S7).

## Discussion

Our main finding is that mechanosensitive channel Piezo1 could coordinate the crosstalk between MSCs and T cells under mechanical loading, to regulate the bone remodeling process in OA. Specifically, the MSCs in subchondral bone sense mechanical loading to recruit inflammatory T cells, especially Th17 cells to activate the inflammatory response. Piezo1 has been reported to act in osteoblast lineage cells as a regulator of bone remodeling *via* sensing mechanical loading [34, 35]. Piezo1-mediated mechanotransduction enhances pathological new bone formation at the entheses in ankylosing spondylitis (AS). AS-associated inflammatory cytokines were found to enhance Piezo1 expression [36]. Yoda1 activates Piezo1 to alleviate bone loss in mouse models of ovariectomy and aging, which is a potential therapeutic target for osteoporosis [37]. In view of its key effect, it is very important to explore the role of Piezo1 in osteoarthritis.

Evidence suggests that the interaction between inflammation and bone remodeling is affected by the biomechanical environment during bone-fracture repair and regeneration. The molecular events underlying these interactions, however, are not fully understood [38]. Here we present the first evidence that Piezo1 controls the interaction of MSCs and Th17 cells during bone remodeling under biomechanical environment. We also detected increased expression of Piezo1 in human temporomandibular joint OA cartilage and bone, which is similar to previous reports that increased expression of Piezo1 in chondrocytes under high mechanical stress [39]. Next, we analyzed the role of Piezo1 in bone metabolism using *Wnt1*^*cre*^*; Piezo1*^*fl/fl*^ mice, and the results revealed that *Wnt1cre; Piezo1*^*fl/fl*^ mice showed a slight bone density decrease in condyle in ground condition. Furthermore, *Wnt1cre; Piezo1*^*fl/fl*^ mice showed less bone deterioration and cartilage degradation induced by mechanical loading, which led to obvious bone abrasion in control mice. It has been reported that Piezo1 is a skeletal mechanosensor that controls bone homeostasis. Loss of Peizo1 specifically in osteoblastic cells (*Prx1Cre; Piezo1*^*fl/fl*^ mice), but not osteoclastic *Piezo1* deficiency (*CtskCre; Piezo1*^*fl/fl*^ mice) demonstrated decreased bone density in femur with decreased BV/TV and Tb.N. However, *Prx1Cre; Piezo1*^*fl/fl*^ mice did not exhibit tail suspension–induced bone loss [35]. Moreover, Piezo1 failed to alter bone metabolism in the skull in ground conditions [29]. These data are consistent with our reports, which support the hypothesis that Piezo1 in osteoblastic cells is required to sense mechanical loading and regulate bone metabolism.

Our results revealed that the inflammatory cytokines IFN-γ and IL-17 were enriched under mechanical loading in the TMJOA group. Recently evidence suggests that Piezo1 in CD4^+^ T cells restrains regulatory T cells in the context of an autoimmune neuroinflammation model [23, 40]. Thus, we analyzed whether T cells directly sense the mechanical loading to initiate and activate the inflammatory response using *CD4*^*+*^*cre, Piezo1*^*fl/fl*^ mice and found that Piezo1 deletion in CD4^+^ T cells failed to alter the bone resorption and cartilage degeneration under mechanical loading. Moreover, differentiation of naïve CD4^+^ T cells into Th1, Th17 effector cells, and Treg cells in vitro proceeded normally in the absence of Piezo1, which is consistent to the previous report [23]. Mechanical force stimulation also failed to alter the polarization of naïve CD4^+^ T cells into Th1, Th17, and Treg cells in vitro. We further demonstrated the comparable cellularity of lymphoid organs in *CD4*^*+*^*cre, Piezo1*^*fl/fl*^ mice, suggesting that immune homeostasis functions of T cells remained intact. However, it’s reported the elevated frequencies of Treg cells were detected during experimental autoimmune encephalomyelitis (EAE) in vivo. However, the Piezo1-deficient Treg cells developed normally and showed similar suppressive capacity compared with wild-type controls. These results suggested that Treg cells could be regulated by Piezo1 only when multiple cellular components are challenged during a strong autoimmune response, such as EAE [23]. Our results imply that Piezo1 is dispensable for CD4^+^ T cell property and response at least under a biomechanical environment directly. However, the underlying mechanism for the difference between the response process of Piezo1 under mechanical stimulus and strong autoimmune response needs further investigation.

The inflammatory T cells were recruited by MSCs after sensing mechanical stimulus, consequently promoting Th17 cells polarization by secreting MIF. Moreover, IL-17 knockout mice showed less bone resorption and cartilage thinning in TMJOA. Evidence has shown that inflammatory cytokines IL-17 and IL-6 are increased in TMJOA [14]. To our knowledge, this is the first verification that IL-17 controls the development of TMJOA directly using knockout mice. Moreover, our results revealed significant upregulation of MIF expression in the OA model as well as increased MIF levels under mechanical loading in MSCs. The increased MIF secretion after mechanical stimulus was found to recruit T cells and promote Th17 polarization to initiate the inflammatory response in OA. Research on the role of MIF in OA is limited and has yielded contradictory results [41]. It was found that gene knock-out of MIF could reduce the severity of OA in aged mice [42]. However, the results also indicated that there were no preventive effects on destabilization of the medial meniscus-induced osteoarthritis in young mice treated with MIF neutralizing antibody [43]. Our results showed that the local injection of MIF neutralizing antibody could partially attenuate the bone deterioration in TMJOA, highlighting its potential as a promising therapeutic strategy for TMJOA treatment. Further research is needed to explore the specific mechanisms and optimize the application of MIF-neutralizing antibodies for the treatment of TMJOA. It has been reported that MIF enhances type 3 immunity by promoting T regulatory cells (Treg) in humans and mice to obtain a Th17 cell-like phenotype during the onset of spinal arthritis, including up-regulating IL-17 and IL-22 in vitro [8]. MIF may directly bind to HVEM, and up-regulated HVEM following activation of NF-κB signaling to promote Th17 cell differentiation in patients of Hashimoto’s thyroiditis [44]. Moreover, MIF can regulate Th17 cell differentiation and participate in the pathogenesis of benign lymphoepithelial lesions through MAPK and PI3K/Akt pathways [45]. Our results demonstrated that MIF can indeed promote the differentiation of Th17 cells. However, the specific mechanism needs further study.

Mechanical stimulus resulted in a decrease in MSC stemness, including inhibited cell proliferation and decreased osteochondrogenic differentiation. Importantly, the impairment of MSC stemness caused by mechanical loading could be partially restored by Piezo1 siRNA treatment. Furthermore, we revealed that mechanical force could induce the Ca^2+^ influx *via* Piezo1 channel, resulting in a direct interaction between CamKII and STAT1 that had been phosphorylated at Ser727. STAT1-dependent induction of nicotinamide phosphoribosyl transferase participates in the metabolic shifts of IFN-γ-activated macrophage [46]. The STAT1 signaling pathway controls the inflammation-triggered metabolic skewing of MSCs to influence its immunomodulatory capacity [47, 48]. However, the role of STAT1 in the property of MSCs under mechanical stress remains unclear. In the present study, we demonstrated that mechanical stimuli activate the STAT1 pathway, promoting HK2-mediated glycolysis in MSCs. Mechanically, we discovered that STAT1 directly binds to the promoter region of HK2, suggesting a direct regulatory role. This interplay activates the phosphorylation of STAT1 to upregulate HK2-mediated glycolysis, thereby promoting the secretion of MIF by MSCs after mechanical loading. To our knowledge, this is the first report to link mechanical force with STAT1 signaling to control bone metabolism. Mechanical force can also activate the Alk5-Shc pathway [49] and TGF-β pathway [50]. Further investigation is needed to determine the involvement of these pathways in OA and glycolysis.

Our study revealed that mechanical stimulus significantly increased HK2 expression in MSCs. Knockdown of HK2 attenuated the increased glycolysis of MSCs induced by mechanical loading. Furthermore, HK2 inhibition restored the functional capacity of MSCs impaired by mechanical loading. Moreover, HK2 orchestrates the intricate interplay between MSCs and Th17 cells by modulating MIF expression and T-cell recruitment. MSCs are multipotent cells that serve as precursors to various cell types, including osteoblasts, chondrocytes, and adipocytes [43]. These results indicate that mechanical stimulation can regulate the directionality of MSC differentiation and determine their fate [51]. Previous studies have reported elevated expression of pyruvate kinase M2 in OA chondrocytes [52]. Under inflammatory conditions, chondrocytes undergo metabolic reprogramming that favors glycolysis through NF-κB activation. LDHA promotes the formation of reactive oxygen species in chondrocytes during inflammation, and inhibiting LDHA activity has shown potential for attenuating OA [53, 54]. To our knowledge, this study is the first to report the role of HK2-mediated glycolysis in TMJOA. Notably, HK2 inhibition by 3-BP showed promising results in attenuating bone abrasion and cartilage degeneration in OA. These findings suggest that glycolysis is a potential therapeutic target for OA. 3-BP has already demonstrated efficacy as a general anticancer agent and is currently undergoing phase II clinical trials [55]. Further studies are necessary to optimize the application of 3-BP in OA treatment. These findings highlight the potential of targeting glycolysis, specifically HK2, as a therapeutic strategy for OA. It has been reported that superoxide dismutase 2 increases the glycolysis level and promotes the immunosuppression capacity of MSCs at the expense of adipocyte differentiation [56]. MSCs can undergo high-level glycolysis in an inflammatory environment, and interfering with glycolysis can affect the up-regulation of indoleamine 2,3-dioxygenase and inhibit its immunomodulation [47]. In our study, we found that the increase in glycolysis of MSCs after mechanical stimulation was related to the IL-17 signaling pathway, as assessed by RNA-sequencing analysis. Moreover, MIF secreted by MSCs upon mechanical treatment could recruit T cells and promote Th17 differentiation.

It has been reported that glucose metabolism switches to glycolysis in fibroblast-like synoviocytes and chondrocytes, which is related to the progression of osteoarthritis and rheumatoid arthritis [57], characterized by increased secretion of proinflammatory cytokines and bone erosion [58]. Our results were consistent with these reports and demonstrated that the glycolysis of MSCs increased after sensing mechanical cues in OA, leading to increased MIF secretion and bone erosion. This in situ interaction of MSCs with T cells differs from that of MSCs grown in culture and subsequently used for infiltration to suppress. However, the underlying mechanism requires further investigation. The activation of hypoxia-inducible factor 1 (HIF-1) is closely related to cellular energy metabolism, especially glycolysis. Aerobic glycolysis mediated by mTOR and HIF-1 is the metabolic basis of trained immunity [59]. Inhibition of HIF-1-mediated aerobic glycolysis can inhibit osteoclast differentiation, thus slowing down bone erosion in OA. Our RNA sequencing results are also consistent with the above research [60].

In summary, our findings reveal that Piezo1 coordinates mechanical signals and bone inflammation *via* crosstalk between MSCs and Th17 cells. Specifically, Piezo1 senses mechanical force and activates HK2-mediated glycolysis through the STAT1 signaling pathway, driving bone inflammation *via* MIF in OA. These results provide novel insights into the regulatory mechanisms of mechanical stress-mediated OA and suggest potential therapeutic targets for OA treatment.

## Materials and methods

### Experimental animals

Female Sprague–Dawley rats (6-8 wk-old; 140–160 g) and male mice (8–10 wk old; 20–22 g) were purchased from Vital River Laboratory Animal Technology (Beijing, China). *CD4 cre* and *IL-17*^*−/−*^ C57BL/6 mice were kindly provided by Dr Chen Dong (Tsinghua University School of Medicine, Beijing, China). Wnt1-cre mice (JAX, http://jaxmice.jax.org/strain/003829) and ROSA26-tdTomato mice (JAX, https://www.jax.org/strain/007914) were provided by Dr Hu Zhao in Chinese Institute for Brain Research, Beijing. *Piezo1*^*flox/flox*^ mice (JAX, https://www.jax.org/strain/029213) were provided by Dr. Bo Shen in National Institute of Biological Science, Beijing. The *CD4cre; Piezo1*^*fl/fl*^ and Wnt1*cre*; *Piezo1*^*fl/fl*^ mice were generated by crossing CD4*cre*, Wnt1*cre*, and *Piezo1*^*flox/flox*^ mice respectively. The primers for identifying were as follows: *Piezo1* ^*flox*^ (wild type: 188 bp; mutant: 380 bp): Forward primer 5’-GCCTAGATTCACCTGGCTTC-3’, Reverse primer 3’- GCTCTTAACCATTGAGCCATCT-5’; Wnt1 ^cre^ (mutant: 475 bp): Forward primer 5’- CAGCGCCGCAACTATAAGAG-3’, Reverse primer 3’ -CATCGACCGGTAATGCAG-5’; tdTomato (mutant: 196 bp): Forward primer 5’- CTGTTCCTGTACGGCATGG-3’, Reverse primer 3’- CCTATGCGACGAAATTACGG-5’. In this study, the random number table method was used to determine how to assign animals to the experimental group and deal with them. Animals are cared for according to the institutional guidelines set by the University Ethics Committee. Protocols were approved by the Animal Care and Use Committee of the Health Science Center, Peking University (No. LA2021488).

### Antibodies

HK2 (Abcam, ab209847), LDHA (Abcam, ab47010 (Abcam, ab47010), p-JAK1 (Cell Signaling Technology, 74129), p-JAK2 (Cell Signaling Technology, 3776,), JAK1 (Cell Signaling Technology, 3332), JAK2 (Cell Signaling Technology, 3230), p-STAT1-S727 (Abcam, ab278718), p-STAT1-Y701 (Abcam, ab30645), STAT1 (Cell Signaling Technology, 14995), Piezo1(Abcam, ab128245), CAMKII(Santa, sc-5306)IL-17(Abcam, ab302923), IFN-γ(Invitrogen, PA5-119649), MIF (Biolegend, 524001), ALP (Abcam, ab15411), RUNX2 (Cell Signaling Technology, 8486), SOX9 (Abcam, ab27414), ACAN (Sigma, MABT83) and β-actin (ZSGB-bio, TA09).

### The TMJOA animal model

Rats were randomly divided into four groups (*n* = 5): sham-operated control group, TMJOA group, 3-BP group (the HK2 inhibitor), and anti-MIF group (the MIF neutralizing antibody). After anaesthetization, a metal tube (length = 4.5 mm, inner diameter = 3.5 mm) was affixed to the left mandibular incisor. The tube’s tip was bent to a 135° angle, which tilted the labial side and caused the upper and lower incisors to bite in a cross-bite relationship. The tubes were carefully bonded with zinc phosphate cement and were checked every other day [61]. Piezo1^*fl*/*fl*^ mice were randomly divided into two groups: a control group and a TMJOA group. Wnt1*cre*; Piezo1^*fl*/*fl*^ mice/ CD4 *cre*; Piezo1^*fl*/*fl*^ mice were also randomly divided into the control group and TMJOA group. Regarding the UAC model of mice, the metal tube (length = 1.5 mm, inner diameter = 0.61 mm, thickness = 0.3 mm) was used, the remaining procedures were the same as the rat model. No detachment of the metal tube was found during the entire experimental period. Control rats were subjected to the same procedure, but no metal tube was adhered. In the 3-BP group, 10 mg/kg 3-BP was injected intraperitoneally every three days for two weeks [62]. In the anti-MIF group, 10 μL MIF neutralizing antibody was injected into the temporomandibular joint cavity every three days for two weeks. Rats were sacrificed to harvest samples at 2 weeks for further evaluation.

### Patient samples collection

Clinical diagnosis and sample collection were obtained from the Department of Surgery, School and Hospital of Stomatology, Peking University. The clinical diagnosis, imaging data, and resected disused condylar tissue were collected. The patients underwent hemi-mandibular resection because of the benign or malignant tumors of the jaw. The condyles were collected from the resected specimens according to the pathological diagnosis. The intact condyles not involved by the tumors were collected and were divided into normal and TMJOA groups by imaging and pathological diagnosis for further evaluation (Supplementary Table 2). This study was approved by the Ethics Committee School and Hospital of Stomatology, Peking University (2022-12-83-07). The written informed consent was obtained from the patients.

### Isolation and culture of subchondral MSCs and mechanical stimulation

Mesenchymal stem cells (MSCs) from the condylar subchondral bone of 6-8week rats were isolated and cultured as previously reported. Briefly, we obtained a small piece of condylar subchondral bone tissues. Then the bone was carefully sectioned using a scalpel and subsequently digested with 3 mg/mL collagenase type I and 4 mg/mL dispase II (Roche Diagnostic, Indianapolis, IN, USA) [63]. Then, the cell suspension was cultured with the α-MEM complete culture medium supplemented with 15% of heat-inactivated fetal bovine serum (FBS), 100 µg/mL Gentamicin, and 2 mM L-glutamine (all from Gibco, Life Technologies, Carlsbad, CA) referred to as cell culture media. The stem cell markers of MSCs isolated from subchondral bone were identified by flow cytometry analysis. The MSCs in passages 3–5 were used for further experiments. For the RNA-seq experiments, the human jawbone MSCs were used and were provided by ORAL STEM CELL BANK run by Beijing Tason Biotech Co, Ltd).

For mechanical stimulation, MSCs (1 × 10^5^ cells/well) were seeded into a 6-well plate under the culture medium for 12 h at 37 °C in 5% CO_2._ Then the MSCs were added a compressed continuous force described previously [64]. Briefly, a cover glass was placed on the monolayer MSCs with the force adjusted by lead granules of 2.5 g/cm^2^ for 12 h to evaluate the effects. The concentration of 3 bp treated cells was 80μm [65].

### T cells polarization

CD4^+^CD25^−^CD62L^hi^CD44^low^ naïve T cells were isolated from the spleen and peripheral lymph nodes of C57BL/6J mice and activated by plate-bound anti-CD3 and anti-CD28 (5 μg/ml). Anti-CD4 (eBioscience, 17-0042-83), anti-CD25 (eBioscience, 25-0251-82), anti-CD62L (eBioscience, 47-0621-82), and anti-CD44 (eBioscience, 48-0441-82) antibodies were used to label cell surface makers. Th1 cells were induced with the combination of mouse interleukin 2 (mIL-2) (25U/ml) and mouse interleukin 12 (mIL-12) (15 ng/ml). Th17 cells were induced with the combination of human TGF-β (1 ng/ml), and mouse interleukin 6 (mIL-6) (20 ng/ml). Treg cells were induced with the combination of human TGF-β (2 ng/ml), and mouse interleukin 2 (mIL-2) (25U/ml). Four days later, the cells were collected for further analysis. Anti-CD3 and anti-CD28 antibodies were purchased from Bio X Cell, human TGF-β were from the R&D System, and other cytokines were from PeproTech.

To detect intracellular cytokines, cells were stimulated in with phorbol 12-myristate 13-acetate (PMA; 50 ng/ml; Sigma-Aldrich), ionomycin (500 ng/ml; Sigma-Aldrich), and GolgiStop (BD Biosciences) for 4 hours at 37 °C in T cell culture medium. Cells were first labeled with Fixable Viability Dye (Invitrogen, 65-0864-18) in PBS for 30 min to eliminate dead cells from analysis. Cells were washed, fixed, and permeabilized using Foxp3 staining buffer set (eBioscience, 00-5523-00). The following antibodies were used to detect intracellular markers: anti–IL-17A (eBioscience, 48-7177-82), anti-IFN-γ(eBioscience, 48-7311-82), anti-Foxp3 (eBioscience, 48-5773-82), and anti-Ki67 (BD Biosciences, 556027). Data were acquired using BD LSRFortessa (BD Biosciences) flow cytometers. Fluorescence intensity was plotted against forward scatter area or height (FSC-A or FSC-H), and analyzed using FlowJo (version 10.8.1) analysis software (FlowJo LLC, Ashland, Oregon).

### RNA-sequencing

RNA-sequencing analyses of total RNA from control and force-treated MSCs were performed at the Beijing Genomics Institution. Three RNA samples from each group were used for RNA-seq analysis. For each sample, we used NEBNext Ultra RNA library Pre Kit to prepare a sequencing library from 1 μg of total RNA, and 2 × 100 paired-end sequencing in fat run mode was performed using the HiSeq 2500 and Illumina TruSeq SBS-Kit v2 (200 cycles). Illunina’s bcl bcl2fastq v1.8.4 software (Illumina, San Diego, CA) was used to convert the resulting base calling (.bcl) to FASTQ files. Mapping RNA-seq reads on the human genome (GRCh38/hg38) after trimming the adaptors, transcript assembly, and abundance estimation were performed using DNASTAR Lasergene v15.0 (DNASTAR, Madison, WI) and reported using FPKM (fragments per kilobase of exon per pillion fragments mapped). Genes term with a P-value < 0.05 and fold change > 2 were considered statistically significant. The different genes between the two groups are listed in Supplementary Table 1. The enrichment of gene clusters and Kyoto Encyclopedia of Genes and Genomes (KEGG) pathways based on different genes between two groups were performed using Gene Set Enrichment Analysis (GSEA). The RNA seq data was deposited in the GEO database (GSE: GSE247273).

### Glucose Consumption and Lactate Production Measurement

Glucose consumption was determined by a glucose assay kit (Sigma, St. Louis, MO, USA) following the manufacturer’s instructions. Briefly. After collecting the medium, the samples (10 μL) and glucose assay reagent (1 ml) were mixed at room temperature for 15 minutes. The absorbance was measured at 340 nm in a microplate reader.

Lactate levels were measured by a lactate colorimetric/fluorometric assay kit (BioVision, Mountain View, CA, USA) according to the manufacturer’s instructions. 2 µl culture medium was added to a 96-well plate and then the lactate assay buffer was used to adjust the total volume to 50 µl/well. For each well, a total of 50 µl reaction mix with 46 μl lactate assay buffer, 2 µl lactate enzyme mix, and 2 µl probe was added. Then the samples were incubated for 30 min at room temperature in the dark, followed by measuring the absorbance (OD 570 nm) in a microplate reader.

### ECAR measurements with Seahorse assay

For Seahorse (Agilent) tests, MSCs were plated in XF96 cell culture microplates (Agilent) at a density of 1 × 10^4^ cells/well. For the glycolysis assay, MSCs were cultured in a CO_2_-free incubator at 37 °C using hippocampal XF assay medium free of glucose prior to analysis. Then the basal level of ECAR, and ECAR stimulated by glucose (10 mM), oligomycin (1 μM), and 2-deoxyglucose (2-DG; 50 mM) were measured using the Seahorse XFe96 Analyzer.

### Osteogenic differentiation

MSCs were first plated in 6-well plates at a density of 2 × 105 cells/well. MSCs were cultured in osteogenic medium containing 0.1 mM β-mercaptoethanol, 10 m M β-glycerophosphate, 1 nM dexamethasone, and 50 μg/mL ascorbic acid in growth medium. After 7 days of osteogenic induction, alkaline phosphatase (ALP, Thermo Fisher Scientific) staining was performed, and the protein and RNA were collected to detect the expression level of RUNX2 and ALP. After 21 days of induction, the mineralized nodule formation was analyzed with 1% Alizarin Red S staining (Sigma). The ALP/Alizarin Red-positive area was measured using ImageJ software and shown as the percentage of Alizarin Red-positive area over the total area.

### Chondrongenic differentiation

For chondrogenic differentiation, MSCs (2 × 10^5^ cells/well) were cultured in chondrongenic medium containing 1% ITS + , 0.1 uM Dexamethasone sodium phosphate, 2 mM Sodium pyrubate, 0.1 mM L-ascorbic acid phosphate in growth medium. After 7 days of induction, the protein and RNA were collected to detect the expression level of SOX9 and ACAN. After 21 days of induction, the cartilage formation was analyzed with Alcian blue staining (Solarbio) at room temperature. The Alcian blue-positive area was measured using ImageJ software and shown as the percentage of the Alcian blue-positive area over the total area.

### Quantitative real-time Polymerase Chain Reaction (PCR)

Total RNA was isolated from cell lysate with Trizol reagent (Invitrogen, Carlsbad, CA). The Superscript III Reverse Transcriptase (RT) kit (Invitrogen) we used to synthesize the complementary DNA (cDNA). qPCR was performed using SYBR Green Supermix (Bio-Rad, Hercules, CA, USA) with gene-specific primers. The primers used were listed in appendix Supplementary Table 3. The level of mRNA expression for each gene was normalized to glyceraldehyde 3-phosphate dehydrogenase (GAPDH and Actin).

### Western blot

Total proteins were lysed by RIPA (within 1% PMSF). The 30 μg protein in each group was loaded and separated by SDS-PAGE gel and transferred to a PVDF membrane (Trans-Blot Turbo Transfer Pack, Bio-Rad Hercules, CA). Then, 0.1% Tween-20 and 5% non-fat dry milk were provided to block the membranes for 1 h. The membranes were incubated with primary antibodies (1:1000) overnight at 4 °C followed by HRP-conjugated secondary antibody (1:5000) incubation for 1 h at room temperature. SuperSignal™ West Pico PLUS was used to detect the immunoreactive proteins. The relative intensity of proteins was quantified with ImageJ (NIH).

For Co-Immunoprecipitation assay, the cell lysates were incubated overnight at 4 °C with gentle agitation with the specified antibodies. After that, protein A/G plus Agarose was added and incubated for 2 hours at 4 °C. The immunoprecipitates were obtained by centrifugation at 3,000 g for 5 minutes. After washing with the wash buffer three times, the samples were mixed with SDS loading buffer and heated at 100 °C for 10 minutes. Finally, the proteins were analyzed by western blot.

### GST-pull down

GST-pulldown assay was performed with STAT1-GST Fusion Protein (ProteinTech, Wuhan, China) and Recombinant CAMK2D-His protein (Solarbio, Beijing, China) using GST-pull-down Kit (IK-2004) (Biolinkedin, Shanghai, China). The general procedure involved the following steps: 5 μg of STAT1-GST Fusion Protein and CAMK2D-His protein were mixed with pre-washed GST-labeled protein beads (GST protein was used in the control group). The mixture was supplemented with GST-pull-down buffer and rotated overnight at 4 °C. The magnetic rack was used to gather the magnetic beads, which were subsequently subjected to wash with 500 μL of GST-pull-down buffer three times. The magnetic beads were collected and subsequently mixed with an SDS loading buffer. The proteins were then analyzed by western blot.

### Chromatin immunoprecipitation (ChIP)

ChIP analysis was performed with 4 × 10^6^ cells per group using the ChIP-IT Express Enzymatic Magnetic Chromatin Immunoprecipitation Kit (Active Motif, Carlsbad, CA) according to the manufacturer’s instructions. Briefly, the cells were cross-linked and used for each immunoprecipitation, and chromatin was sheared with a Branson sonicator. To precipitate DNA-protein complexes, the antibody to STAT1 was used (Cell Signaling Technologies) and IgG was used as an isotype control. Then the precipitated DNA was purified with ChIP DNA Purification Kit (Active Motif) and amplificated using SYBER green with the 10% sheared chromatin as input.

### siRNA transfection assay

For siRNA transfection, MSCs were plated as 2 × 105 cells/well in 6-well plates and treated with 50 nM vehicle, HK2, STAT1, and Piezo1 siRNAs using riboFECT CP Transfection Kit (Ribobio, C10511-05), according to the manufacturer’s instructions.

### Micro–Computed Tomography (CT) Scanning

The TMJs and knee joint were removed and fixed in 10% formalin overnight. These tissues were scanned with a micro-CT system (Inveon MMCT, Berlin, Germany) at 80 kV, 500 µA, and an image voxel size of 18 µm. Mimics 13.1 software (Materialize, Leuven, Belgium) was used for 3D image reconstruction and segmentation. For the TMJs, the region of interest covered the condylar head, and a total of 40 consecutive cross-sectional images from the most superior point of the condylar head were used. The following parameters, including bone volume to total volume ratio (BV/TV, nondimensional), trabecular thickness (Tb.Th, mm), trabecular number (Tb.N, 1/mm), trabecular spacing (Tb.Sp, mm) in each group were measured and analyzed after animal model establishment. For the knee joint, we defined the region of interest as the whole subchondral bone. We used a total of 10 consecutive images from the medial tibial plateau for 3D reconstruction and analysis. The following parameters in each group were measured and compared 4 weeks after operation.

### Immunohistochemistry and immunofluorescence staining

For immunohistochemistry, sections were dewaxed and incubated with 5% bovine serum albumin and 0.1% Triton X-100 for 1 h. Then the samples were incubated with primary antibody (Anti-HK2 [1:100], anti-LDHA [1:200], anti-phosphorylated (p)STAT1 [1:100] anti- STAT1 [1:100], anti-MIF [1:100], anti-CD90 [1:100], anti-CD90 [1:100], anti-IL-17 [1:100] and anti-IFN-γ [1:100] and anti-ALP [1:200]) overnight at 4 °C followed by the secondary antibody incubation at room temperature for 1 h. Then the images were taken by microscope (Leica, Wetzlar, Germany). Positive areas were measured with Image-Pro Plus software 6.0 (Media Cybernetics, Rockville, MD, United States).

For immunofluorescence staining, the sections were treated with 0.1% Triton at room temperature for 30 min and non-specific antigen binding sites were blocked with 5% bovine serum albumin. Then the primary antibodies were incubated overnight at 4 °C. Then the samples were treated with Alexafluoro 488 or Alexafluoro 568 conjugated secondary antibody (1:200, Invitrogen) for 1 h at room temperature. Finally, the slides were mounted with mounting medium containing 4’,6-diamidino2-phenylindole (DAPI). All sections were observed with a Leica fluorescence microscope.

For tartrate-resistant acid phosphatase (TRAP) staining, the sections were stained using a leukocyte acid phosphatase kit (387A-1KT, Sigma-Aldrich, St. Louis, MO) to detect the osteoclasts.

### Statistical analysis

Statistical analysis was performed with SPSS 22.0. All data were presented as mean ± SD. The comparison between two groups was analyzed by independent unpaired two-tailed Student’s t-tests, and comparisons among more than two groups were conducted using one-way analysis of variance (ANOVA) with the Bonferroni correction. The comparison related with patient samples was analyzed by Covariance analysis after controlling the covariates (including gender and age). *P*-values < 0.05 were considered as statistical significance.

## Supplementary information

Supplemental materials.pdf

Supplemantal Material-Uncropped western blot images

Supplementary Table 1 The different genes list between Control and Force groups

Supplementary Table 2 The TMJOA samples used in this study.xlsx

Supplementry Table 3 The primers used for qPCR.xlsx

## Data Availability

The RNA seq data was deposited in the GEO database (GSE: GSE247273).

## References

[1] Feng X, McDonald JM. Disorders of bone remodeling. Annu Rev Pathol Mech Dis. 2011;6:121–45. 10.1146/annurev-pathol-011110-130203 PMC3571087 20936937

[2] Vico L, Collet P, Guignandon A, Lafage-Proust M-H, Thomas T, Rehailia M, et al. Effects of long-term microgravity exposure on cancellous and cortical weight-bearing bones of cosmonauts. Lancet. 2000;355:1607–11. 10821365 10.1016/s0140-6736(00)02217-0

[3] Wang L, You X, Zhang L, Zhang C, Zou W. Mechanical regulation of bone remodeling. Bone Res. 2022;10:16. 35181672 10.1038/s41413-022-00190-4 PMC8857305

[4] Findlay DM, Kuliwaba JS. Bone–cartilage crosstalk: a conversation for understanding osteoarthritis. Bone Res. 2016;4:16028. 27672480 10.1038/boneres.2016.28 PMC5028726

[5] Hosseini SM, Wilson W, Ito K, van Donkelaar CC. A numerical model to study mechanically induced initiation and progression of damage in articular cartilage. Osteoarthr Cartil. 2014;22:95–103. 10.1016/j.joca.2013.10.010 24185112

[6] Zhu X, Chan YT, Yung PSH, Tuan RS, Jiang Y. Subchondral bone remodeling: a therapeutic target for osteoarthritis. Front Cell Dev Biol. 2020;8:607764. 33553146 10.3389/fcell.2020.607764 PMC7859330

[7] Wang XD, Zhang JN, Gan YH, Zhou YH. Current understanding of pathogenesis and treatment of TMJ osteoarthritis. J Dent Res. 2015;94:666–73. 25744069 10.1177/0022034515574770

[8] Nakamura A, Zeng F, Nakamura S, Reid KT, Gracey E, Lim M, et al. Macrophage migration inhibitory factor drives pathology in a mouse model of spondyloarthritis and is associated with human disease. Sci Transl Med. 2021;13. eabg1210. 34669443 10.1126/scitranslmed.abg1210

[9] Sun W, Chi S, Li Y, Ling S, Tan Y, Xu Y, et al. The mechanosensitive Piezo1 channel is required for bone formation. Elife. 2019;8:e47454. 31290742 10.7554/eLife.47454 PMC6685704

[10] Shen B, Tasdogan A, Ubellacker JM, Zhang J, Nosyreva ED, Du L, et al. A mechanosensitive peri-arteriolar niche for osteogenesis and lymphopoiesis. Nature. 2021;591:438–44. 33627868 10.1038/s41586-021-03298-5 PMC7979521

[11] Huang H-M, Han C-S, Cui S-J, Zhou Y-K, Xin T-Y, Zhang T, et al. Mechanical force-promoted osteoclastic differentiation via periodontal ligament stem cell exosomal protein ANXA3. Stem Cell Rep. 2022;17:1842–58. 10.1016/j.stemcr.2022.06.006 PMC9391435 35868309

[12] Yu W, Chen C, Kou X, Sui B, Yu T, Liu D, et al. Mechanical force-driven TNFα endocytosis governs stem cell homeostasis. Bone Res. 2021;8:44. 33384406 10.1038/s41413-020-00117-x PMC7775432

[13] Mousawi F, Peng H, Li J, Ponnambalam S, Roger S, Zhao H, et al. Chemical activation of the Piezo1 channel drives mesenchymal stem cell migration via inducing ATP release and activation of P2 receptor purinergic signaling. Stem Cells. 2020;38:410–21. 31746084 10.1002/stem.3114 PMC7064961

[14] Costa AC, de F, de Sousa LM, Dos Santos Alves JM, Goes P, Pereira KMA, et al. Anti-inflammatory and hepatoprotective effects of quercetin in an experimental model of rheumatoid arthritis. Inflammation. 2021;44:2033–43. 34080090 10.1007/s10753-021-01479-y

[15] Nakamura H, Yoshino S, Kato T, Tsuruha J, Nishioka K. T-cell mediated inflammatory pathway in osteoarthritis. Osteoarthr Cartil. 1999;7:401–2. 10.1053/joca.1998.0224 10419780

[16] Pageon SV, Govendir MA, Kempe D, Biro M. Mechanoimmunology: molecular-scale forces govern immune cell functions. Mol Biol Cell. 2018;29:1919–26. 30088799 10.1091/mbc.E18-02-0120 PMC6232972

[17] Thauland TJ, Hu KH, Bruce MA, Butte MJ. Cytoskeletal adaptivity regulates T cell receptor signaling. Sci Signal. 2017;10:eaah3737. 28270556 10.1126/scisignal.aah3737 PMC5854469

[18] Liu Y, Belkina NV, Park C, Nambiar R, Loughhead SM, Patino-Lopez G, et al. Constitutively active ezrin increases membrane tension, slows migration, and impedes endothelial transmigration of lymphocytes in vivo in mice. Blood. 2012;119:445–53. 22106344 10.1182/blood-2011-07-368860 PMC3257010

[19] Faure S, Salazar-Fontana LI, Semichon M, Tybulewicz VLJ, Bismuth G, Trautmann A, et al. ERM proteins regulate cytoskeleton relaxation promoting T cell-APC conjugation. Nat Immunol. 2004;5:272–9. 14758359 10.1038/ni1039

[20] Feng Y, Brazin KN, Kobayashi E, Mallis RJ, Reinherz EL, Lang MJ. Mechanosensing drives acuity of αβ T-cell recognition. Proc Natl Acad Sci USA. 2017;114:E8204–E8213. 28811364 10.1073/pnas.1703559114 PMC5625899

[21] Liu B, Chen W, Evavold BD, Zhu C. Accumulation of dynamic catch bonds between TCR and agonist peptide-MHC triggers T cell signaling. Cell. 2014;157:357–68. 24725404 10.1016/j.cell.2014.02.053 PMC4123688

[22] Saitakis M, Dogniaux S, Goudot C, Bufi N, Asnacios S, Maurin M, et al. Different TCR-induced T lymphocyte responses are potentiated by stiffness with variable sensitivity. Elife. 2017;6:e23190. 28594327 10.7554/eLife.23190 PMC5464771

[23] Jairaman A, Othy S, Dynes JL, Yeromin AV, Zavala A, Greenberg ML, et al. Piezo1 channels restrain regulatory T cells but are dispensable for effector CD4+ T cell responses. Sci Adv. 2021;7:eabg5859. 34233878 10.1126/sciadv.abg5859 PMC8262815

[24] Dehnavi S, Sadeghi M, Tavakol Afshari J, Mohammadi M. Interactions of mesenchymal stromal/stem cells and immune cells following MSC-based therapeutic approaches in rheumatoid arthritis. Cell Immunol. 2023;393–394:104771. 37783061 10.1016/j.cellimm.2023.104771

[25] Matsuo K, Irie N. Osteoclast–osteoblast communication. Arch Biochem Biophys. 2008;473:201–9. 18406338 10.1016/j.abb.2008.03.027

[26] Shi C, Zhang H, Louie K, Mishina Y, Sun H. BMP signaling mediated by BMPR1A in osteoclasts negatively regulates osteoblast mineralization through suppression of Cx43: BMP signaling in OC regulates OC-OB crosstalk. J Cell Biochem. 2017;118:605–14. 27649478 10.1002/jcb.25746 PMC5813677

[27] Movila A, Ishii T, Albassam A, Wisitrasameewong W, Howait M, Yamaguchi T, et al. Macrophage Migration Inhibitory Factor (MIF) supports homing of osteoclast precursors to peripheral osteolytic lesions. J Bone Min Res. 2016;31:1688–1700. 10.1002/jbmr.2854 PMC5010512 27082509

[28] Xin P, Xu X, Deng C, Liu S, Wang Y, Zhou X, et al. The role of JAK/STAT signaling pathway and its inhibitors in diseases. Int Immunopharmacol. 2020;80:106210. 31972425 10.1016/j.intimp.2020.106210

[29] Romac JM-J, Shahid RA, Swain SM, Vigna SR, Liddle RA. Piezo1 is a mechanically activated ion channel and mediates pressure induced pancreatitis. Nat Commun. 2018;9:1715. 29712913 10.1038/s41467-018-04194-9 PMC5928090

[30] Hu X, li J, Fu M, Zhao X, Wang W. The JAK/STAT signaling pathway: from bench to clinic. Sig Transduct Target Ther. 2021;6:402. 10.1038/s41392-021-00791-1 PMC8617206 34824210

[31] Owen KL, Brockwell NK, Parker BS. JAK-STAT Signaling: A Double-Edged Sword of Immune Regulation and Cancer Progression. Cancers. 2019;11:2002. 31842362 10.3390/cancers11122002 PMC6966445

[32] Chodisetti SB, Fike AJ, Domeier PP, Schell SL, Mockus TE, Choi NM, et al. Serine phosphorylation of the STAT1 transactivation domain promotes autoreactive B cell and systemic autoimmunity development. J Immunol. 2020;204:2641–50. 32253245 10.4049/jimmunol.2000170 PMC9305983

[33] Nair JS, DaFonseca CJ, Tjernberg A, Sun W, Darnell JE, Chait BT, et al. Requirement of Ca 2+ and CaMKII for Stat1 Ser-727 phosphorylation in response to IFN-γ. Proc Natl Acad Sci USA. 2002;99:5971–6. 11972023 10.1073/pnas.052159099 PMC122886

[34] Coste B, Mathur J, Schmidt M, Earley TJ, Ranade S, Petrus MJ, et al. Piezo1 and Piezo2 are essential components of distinct mechanically activated cation channels. Science. 2010;330:55–60. 20813920 10.1126/science.1193270 PMC3062430

[35] Wang L, You X, Lotinun S, Zhang L, Wu N, Zou W. Mechanical sensing protein PIEZO1 regulates bone homeostasis via osteoblast-osteoclast crosstalk. Nat Commun. 2020;11:282. 31941964 10.1038/s41467-019-14146-6 PMC6962448

[36] Chen S, Li Z, Chen D, Cui H, Wang J, Li Z, et al. Piezo1-mediated mechanotransduction promotes entheseal pathological new bone formation in ankylosing spondylitis. Ann Rheum Dis. 2023;82:533–45. 36543525 10.1136/ard-2022-223428

[37] Hu Y, Tian H, Chen W, Liu Y, Cao Y, Pei H, et al. The critical role of the Piezo1/β-catenin/ATF4 axis on the stemness of Gli1 + BMSCs during simulated microgravity-induced bone loss. Adv Sci. 2023;10:2303375. 10.1002/advs.202303375 PMC10646271 37759400

[38] Claes L, Recknagel S, Ignatius A. Fracture healing under healthy and inflammatory conditions. Nat Rev Rheumatol. 2012;8:133–43. 22293759 10.1038/nrrheum.2012.1

[39] Lee W, Nims RJ, Savadipour A, Zhang Q, Leddy HA, Liu F, et al. Inflammatory signaling sensitizes Piezo1 mechanotransduction in articular chondrocytes as a pathogenic feed-forward mechanism in osteoarthritis. Proc Natl Acad Sci USA. 2021;118:e2001611118. 33758095 10.1073/pnas.2001611118 PMC8020656

[40] Liu H, Hu J, Zheng Q, Feng X, Zhan F, Wang X, et al. Piezo1 channels as force sensors in mechanical force-related chronic inflammation. Front Immunol. 2022;13:816149. 35154133 10.3389/fimmu.2022.816149 PMC8826255

[41] Ives A, Le Roy D, Théroude C, Bernhagen J, Roger T, Calandra T. Macrophage migration inhibitory factor promotes the migration of dendritic cells through CD74 and the activation of the Src/PI3K/myosin II pathway. FASEB J. 2021;35:e21418. 33774873 10.1096/fj.202001605R

[42] Rowe MA, Harper LR, McNulty MA, Lau AG, Carlson CS, Leng L, et al. Reduced osteoarthritis severity in aged mice with deletion of macrophage migration inhibitory factor. Arthritis Rheumatol. 2017;69:352–61. 27564840 10.1002/art.39844 PMC5274570

[43] Chamberlain G, Fox J, Ashton B, Middleton J. Concise review: mesenchymal stem cells: their phenotype, differentiation capacity, immunological features, and potential for homing. Stem Cells. 2007;25:2739–49. 17656645 10.1634/stemcells.2007-0197

[44] Liu Z, Li Z, Yan G, Lin C, Luo Y, Ye Y, et al. MIF promotes Th17 cell differentiation in Hashimoto’s thyroiditis by binding HVEM and activating NF-κB signaling pathway. Int Immunopharmacol. 2023;121:110494. 37331297 10.1016/j.intimp.2023.110494

[45] Adzavon YM, Zhao P, Ma J, Zhang X, Zhang X, Zhang M, et al. Macrophage migration inhibitory factor contributes to the pathogenesis of benign lymphoepithelial lesion of the lacrimal gland. Cell Commun Signal. 2018;16:70. 30348174 10.1186/s12964-018-0284-4 PMC6196440

[46] Huffaker TB, Ekiz HA, Barba C, Lee S-H, Runtsch MC, Nelson MC, et al. A Stat1 bound enhancer promotes Nampt expression and function within tumor associated macrophages. Nat Commun. 2021;12:2620. 33976173 10.1038/s41467-021-22923-5 PMC8113251

[47] Jitschin R, Böttcher M, Saul D, Lukassen S, Bruns H, Loschinski R, et al. Inflammation-induced glycolytic switch controls suppressivity of mesenchymal stem cells via STAT1 glycosylation. Leukemia. 2019;33:1783–96. 30679801 10.1038/s41375-018-0376-6

[48] Vigo T, La Rocca C, Faicchia D, Procaccini C, Ruggieri M, et al. IFNβ enhances mesenchymal stromal (Stem) cells immunomodulatory function through STAT1-3 activation and mTOR-associated promotion of glucose metabolism. Cell Death Dis. 2019;10:85. 30692524 10.1038/s41419-019-1336-4 PMC6349843

[49] Mehta V, Pang K-L, Givens CS, Chen Z, Huang J, Sweet DT, et al. Mechanical forces regulate endothelial-to-mesenchymal transition and atherosclerosis via an Alk5-Shc mechanotransduction pathway. Sci Adv. 2021;7:eabg5060. 34244146 10.1126/sciadv.abg5060 PMC8270486

[50] Zhen G, Guo Q, Li Y, Wu C, Zhu S, Wang R, et al. Mechanical stress determines the configuration of TGFβ activation in articular cartilage. Nat Commun. 2021;12:1706. 33731712 10.1038/s41467-021-21948-0 PMC7969741

[51] Kelly DJ, Jacobs CR. The role of mechanical signals in regulating chondrogenesis and osteogenesis of mesenchymal stem cells. Birth Defects Res Pt C. 2010;90:75–85. 10.1002/bdrc.20173 20301221

[52] Zhang B, Chen H, Ouyang J, Xie Y, Chen L, Tan Q, et al. SQSTM1-dependent autophagic degradation of PKM2 inhibits the production of mature IL1B/IL-1β and contributes to LIPUS-mediated anti-inflammatory effect. Autophagy. 2020;16:1262–78. 31500508 10.1080/15548627.2019.1664705 PMC7469634

[53] Arra M, Swarnkar G, Ke K, Otero JE, Ying J, Duan X, et al. LDHA-mediated ROS generation in chondrocytes is a potential therapeutic target for osteoarthritis. Nat Commun. 2020;11. 3427. 32647171 10.1038/s41467-020-17242-0 PMC7347613

[54] Bao C, Zhu S, Song K, He C. HK2: a potential regulator of osteoarthritis via glycolytic and non-glycolytic pathways. Cell Commun Signal. 2022;20:132. 36042519 10.1186/s12964-022-00943-y PMC9426234

[55] El Sayed SM. Enhancing anticancer effects, decreasing risks and solving practical problems facing 3-bromopyruvate in clinical oncology: 10 years of research experience. IJN. 2018;13:4699–709. 30154655 10.2147/IJN.S170564 PMC6103555

[56] Li Y, Wang T, Li X, Li W, Lei Y, Shang Q, et al. SOD2 promotes the immunosuppressive function of mesenchymal stem cells at the expense of adipocyte differentiation. Mol Ther. 2024;32:1144–57. 38310354 10.1016/j.ymthe.2024.01.031 PMC11163202

[57] Masoumi M, Mehrabzadeh M, Mahmoudzehi S, Mousavi MJ, Jamalzehi S, Sahebkar A, et al. Role of glucose metabolism in aggressive phenotype of fibroblast-like synoviocytes: Latest evidence and therapeutic approaches in rheumatoid arthritis. Int Immunopharmacol. 2020;89:107064. 33039953 10.1016/j.intimp.2020.107064

[58] De Oliveira PG, Farinon M, Sanchez-Lopez E, Miyamoto S, Guma M. Fibroblast-like Synoviocytes glucose metabolism as a therapeutic target in rheumatoid arthritis. Front Immunol. 2019;10:1743. 31428089 10.3389/fimmu.2019.01743 PMC6688519

[59] Cheng S-C, Quintin J, Cramer RA, Shepardson KM, Saeed S, Kumar V, et al. mTOR- and HIF-1α–mediated aerobic glycolysis as metabolic basis for trained immunity. Science. 2014;345:1250684. 25258083 10.1126/science.1250684 PMC4226238

[60] Kachler K, Andreev D, Thapa S, Royzman D, Gießl A, Karuppusamy S et al. Acod1-mediated inhibition of aerobic glycolysis suppresses osteoclast differentiation and attenuates bone erosion in arthritis. Ann Rheum Dis 2024; ard-2023-224774. 10.1136/ard-2023-224774 PMC11671873 38964754

[61] Yang H, Wen Y, Zhang M, Liu Q, Zhang H, Zhang J, et al. MTORC1 coordinates the autophagy and apoptosis signaling in articular chondrocytes in osteoarthritic temporomandibular joint. Autophagy. 2020;16:271–88. 31007149 10.1080/15548627.2019.1606647 PMC6984599

[62] Garcia-Carbonell R, Divakaruni AS, Lodi A, Vicente-Suarez I, Saha A, Cheroutre H, et al. Critical role of glucose metabolism in rheumatoid arthritis fibroblast-like Synoviocytes. Arthritis Rheumatol. 2016;68:1614–26. 26815411 10.1002/art.39608 PMC4963240

[63] Yamaza T, Ren G, Akiyama K, Chen C, Shi Y, Shi S. Mouse Mandible contains distinctive mesenchymal stem cells. J Dent Res. 2011;90:317–24. 21076121 10.1177/0022034510387796 PMC3034836

[64] Feng L, Yang R, Liu D, Wang X, Song Y, Cao H, et al. PDL progenitor-mediated PDL recovery contributes to orthodontic relapse. J Dent Res. 2016;95:1049–56. 27161015 10.1177/0022034516648604

[65] Lee AW-M, States DJ. Colony-stimulating factor-1 requires PI3-kinase-mediated metabolism for proliferation and survival in myeloid cells. Cell Death Differ. 2006;13:1900–14. 16514418 10.1038/sj.cdd.4401884

