# Supplementary material for: Mechanical sensing protein PIEZO1 controls osteoarthritis via glycolysis mediated mesenchymal stem cells-Th17 cells crosstalk
Source: Cell Death Dis. 2025 Apr 1;16(1):231. doi: 10.1038/s41419-025-07577-1 (PMC11961634; doi:10.1038/s41419-025-07577-1)
Supplement: Supplementary file 1 — Supplemental materials.pdf [file 41419_2025_7577_MOESM1_ESM.pdf]

Supplementary Materials for

Mechanical sensing protein PIEZO1 controls osteoarthritis *via* glycolysis mediated mesenchymal stem cells-Th17 cells crosstalk

Yikun Zhou[1, 2#], Mingzhao Li[1#], Shuai Lin[1#], Zilu Zhu[1], Zimeng Zhuang[1], Shengjie Cui[1], Liujing Chen[1], Ran Zhang[3], Xuedong Wang[1], Bo Shen[4], Chider Chen[5], and Ruili Yang[1]*

[#]These authors contributed equally
[*]Correspondence to: ruiliyang@bjmu.edu.cn

This file includes:

supplementary Figs 1 to 7
Captions for Supplementary Tables S1 to S3

Other Supplementary Materials for this manuscript include the following as an Excel file:

Supplementary Tables S1 to S3

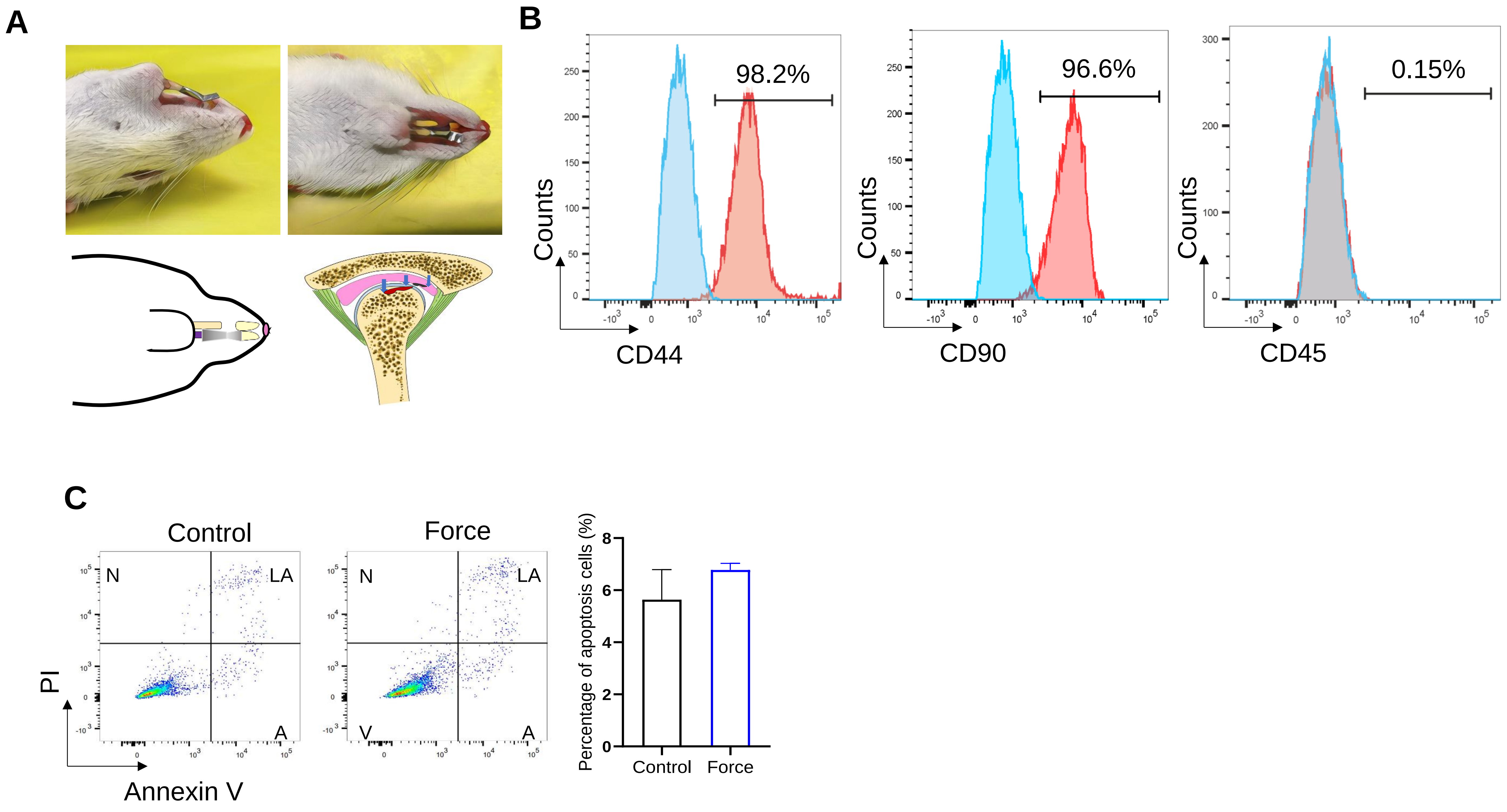

Supplemental Figure 1. Mechanical stimuli lead to osteoarthritis. .(A)Schematic diagram of temporomandibular arthritis animal model. (B) The expression of stem cell surface markers CD44, CD90and CD45 analyzed by flow cytometry.(C)The MSCs apoptosis rate was analyzed by Annexin V/PI detection kit using flow cytometry.Scale bar: 100 μm; data are presented as the mean ± SEM, $^{*}P < 0.05$; $^{**}P < 0.01$; $^{***}P < 0.005$.

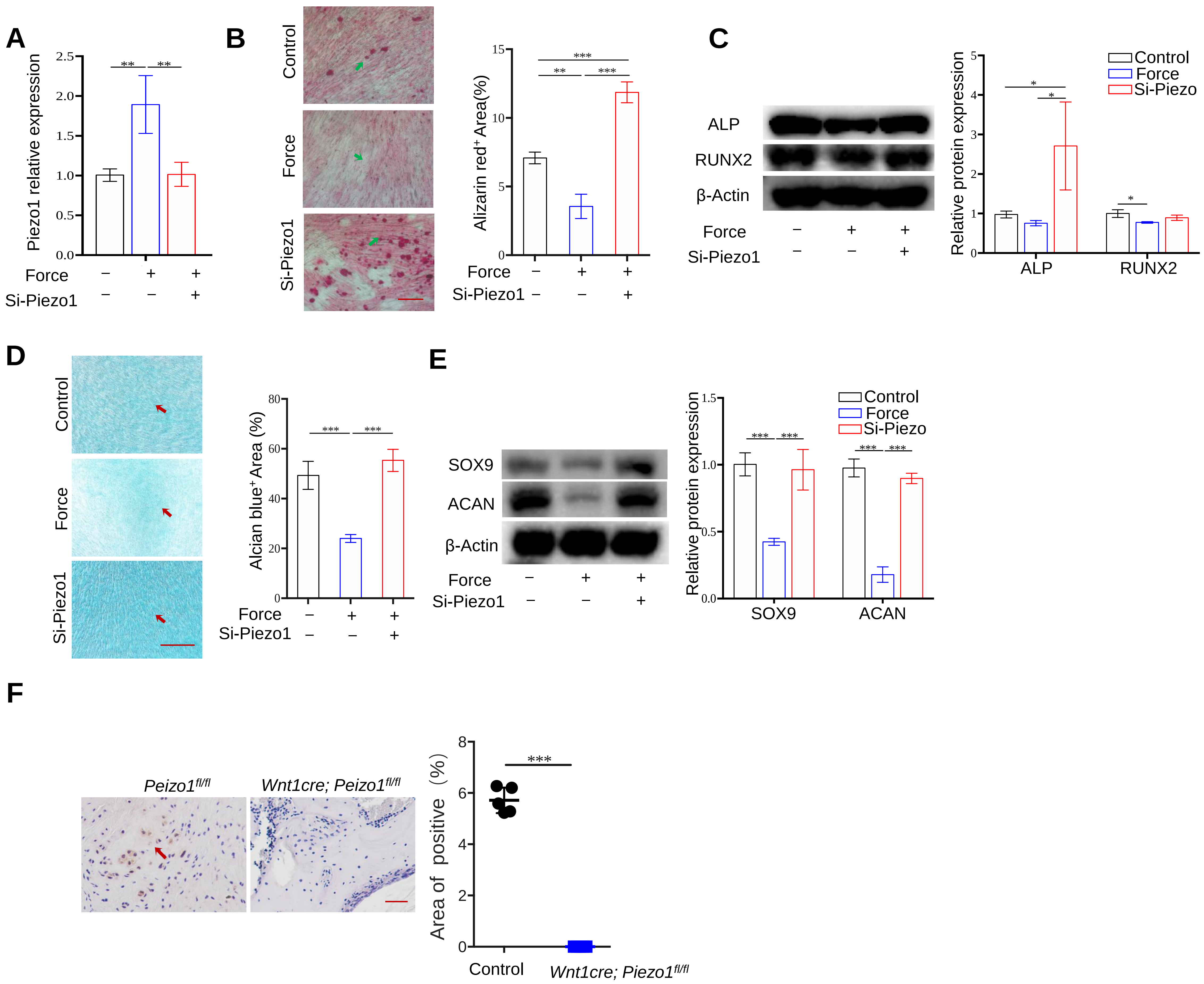

Supplemental Figure 2. Inhibition of Piezo1 partially restored the impaired osteochondrogenic differentiation of MSCs .(A) The expression of Piezo1 of MSCs in control, force with or without Piezo1 siRNA analyzed by qPCR. (B) The osteogenic differentiation capacity of MSCs in control, force with or without Piezo1 siRNA treated groups were analyzed by Alizarin red S staining. (C) The protein expression levels of osteoblast markers ALP and RUNX2 in control, force with or without Piezo1 siRNA treated MSCs. (D) The chondrogenic differentiation capacity of MSCs in control, force with or without Piezo1 siRNA treated MSCs were assessed by Alcian blue staining after 21 days chondrogenic induction. (E) The protein expression levels of chondrogenic markers SOX9 and ACAN in control, force with or without Piezo1 siRNA treated MSCs.(F) The expression of Piezo1 in control and Wnt1cre; Piezo1$^{fl/fl}$ mice condyle subcondral tissues were assessed by immunohistochemical staining. Scale bar: 100 μm; data are presented as the mean ± SEM, $^{*}P < 0.05$; $^{**}P < 0.01$; $^{***}P < 0.005$.

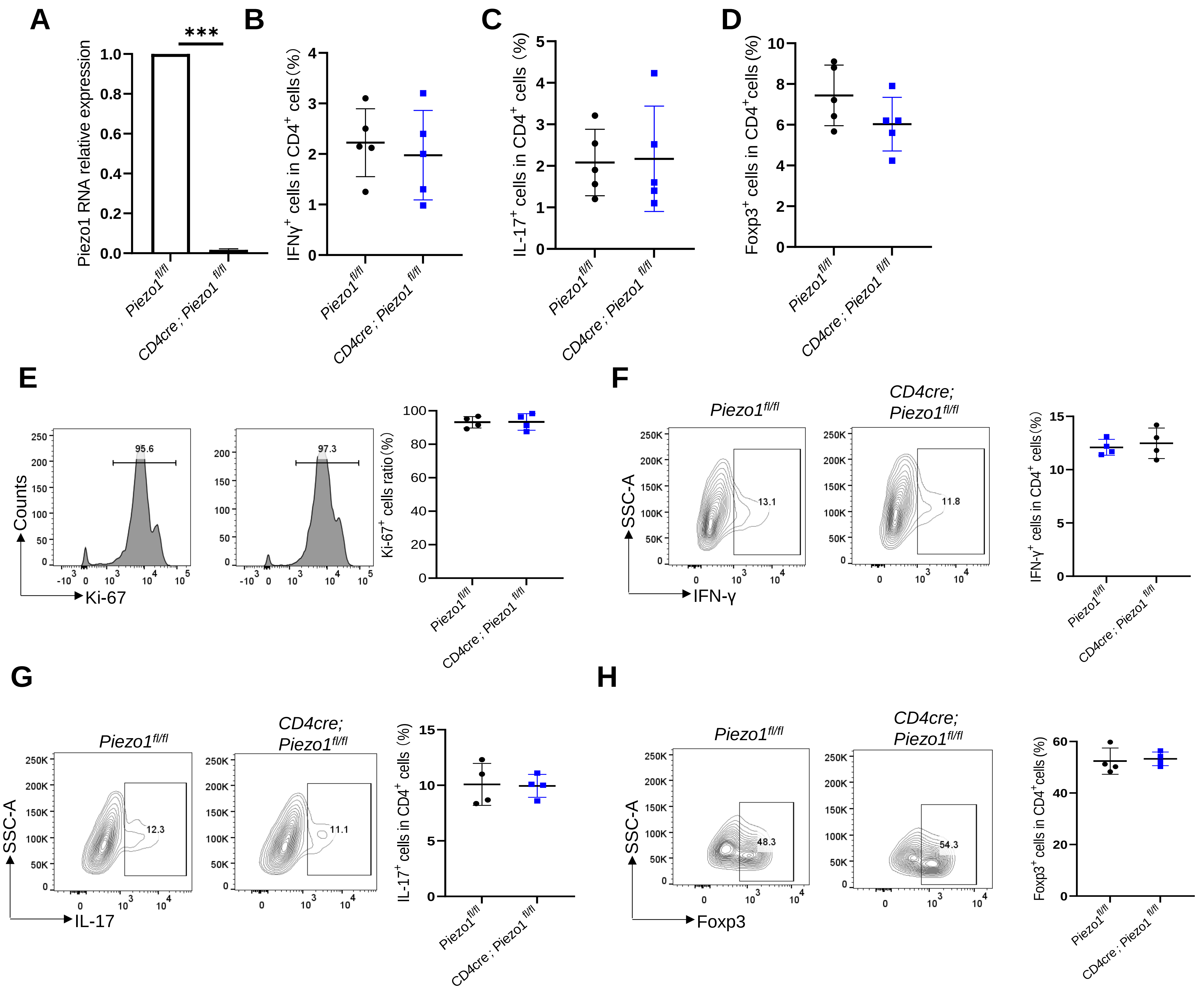

Supplemental Figure 3. Piezo1 in CD4$^+$ T cells failed to control temporomandibular joint OA development.

(A) The gene expression levels of Piezo1 in control and *CD4cre; Piezo1$^{fl/fl}$* mice determined by qPCR. (B-D) T helper cells ratio including Th1, Th17 and Treg cells between control and *CD4cre; Piezo1$^{fl/fl}$* mice.(E)The Ki-67 positive cells ratio in native CD4+ T cells from control and *CD4cre; Piezo1$^{fl/fl}$* mice. (F) IFN-γ positive cells ratio in CD4$^+$ cells between control and *CD4cre; Piezo1$^{fl/fl}$* mice in the polarization induction condition (G) IL-17 positive cells ratio in CD4$^+$ cells between control and *CD4cre; Piezo1$^{fl/fl}$* mice mice in the polarization induction condition .(H) Foxp3 positive cells ratio in CD4$^+$ cells between control and *CD4cre; Piezo1$^{fl/fl}$* mice mice in the polarization induction condition. Data are presented as the mean ± SEM.

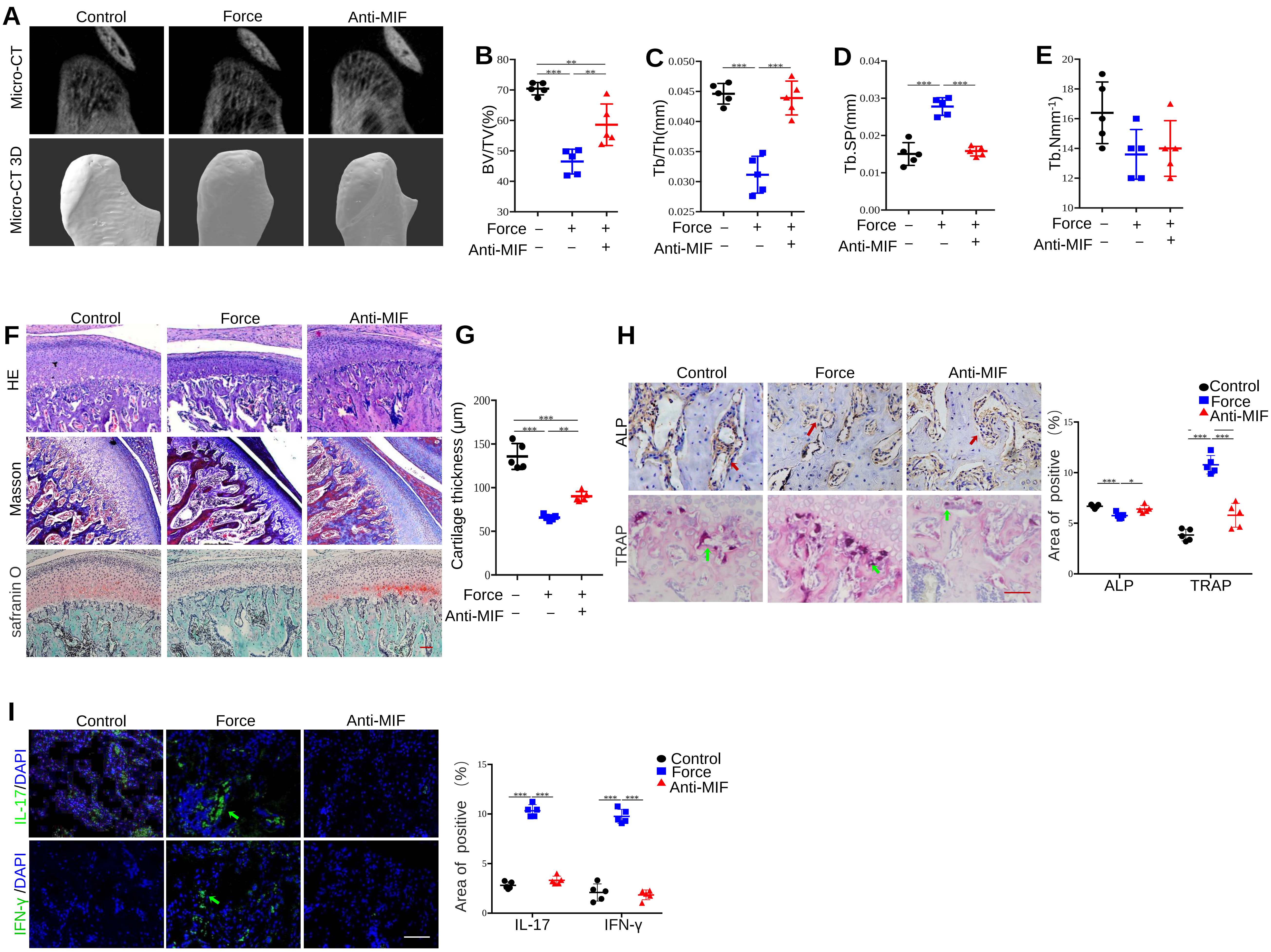

Supplemental Figure 4. Targeting MIF attenuated the bone deterioration of temporomandibular joint OA. (A) The morphology of TMJ condyles in control, force with or without anti-MIF treated groups was shown by micro-CT analysis. (B-E) Quantitative analysis of (B) BV/TV, (C) Tb.Th, (D) Tb.Sp and (E) Tb.N in subchondral bone of TMJ condylar heads determined by micro-CT. (F-G) Characteristics of condyles in control, force with or without anti-MIF treated groups was shown by HE staining, The histological Masson staining and safranin O staining. (H) The ALP positive and TRAP positive cells in control, force with or without anti-MIF treated groups were assessed by ALP staining and TRAP staining. (I) The expression of IL-17 and IFN-γ in control, force with or without anti-MIF treated condyle tissues. TMJ: temporomandibular joint, Scale bar: 100 μm; data are presented as the mean ± SEM, $^{*}P < 0.05$; $^{**}P < 0.01$; $^{***}P < 0.005$.

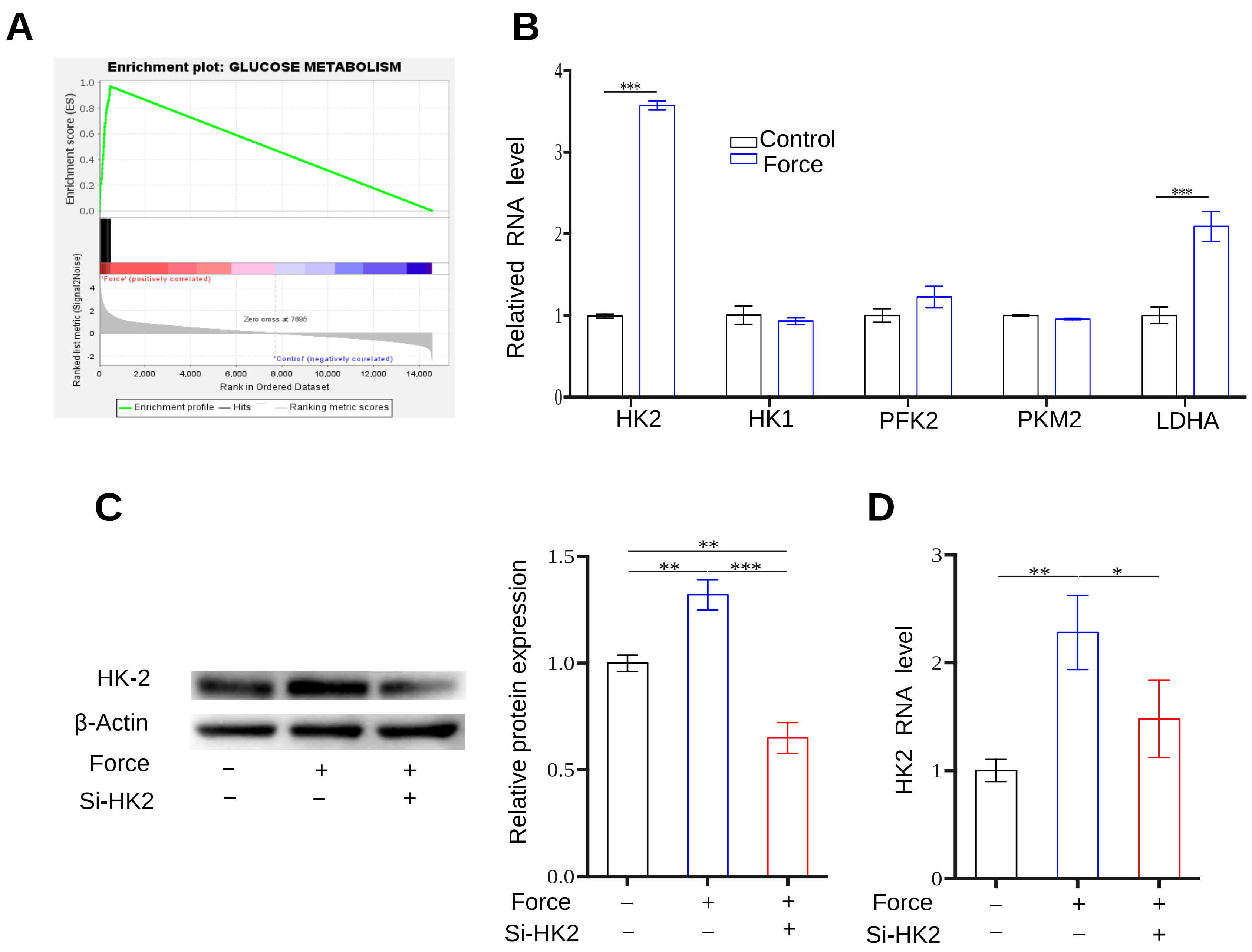

Supplemental Figure 5. HK2-mediated glycolysis alleviated the osteo-chondrogenic differentiation of MSCs.(A) Enrichment plot of glucose metabolism in control and force treated groups analyzed by GSEA. (B) The gene expression levels of glycolysis pathway in control and force treated groups determined by qPCR. (C-D) The protein (C) and gene (D) expression levels of HK2 in control, force with or without SiHK2 treated MSCs. data are presented as the mean ± SEM, **P* < 0.05; ***P* < 0.01; ****P* < 0.005.

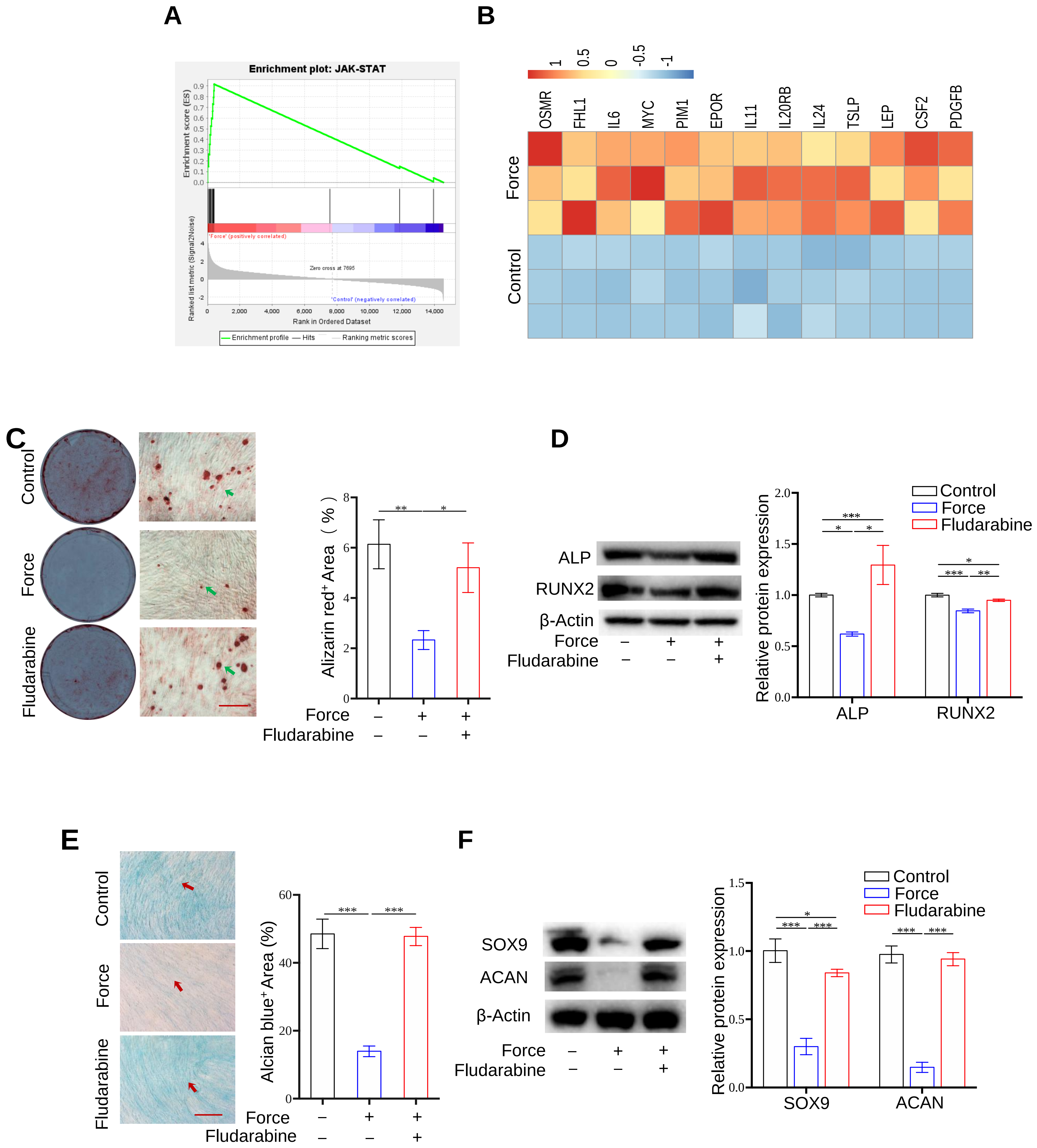

Supplemental Figure 6. Mechanical stimulus activated STAT1 signaling. (A)The enrichment plot of JAK-STAT pathway in control and force treated groups analyzed by GSEA. (B) A heatmap depicting the upregulated and downregulated genes related with JAK-STAT pathway in control and force treated groups. (C) The osteogenic differentiation capacity of MSCs in in control, force with or without fludarabine treated groups were analyzed by Alizarin red S staining. (D) The protein expression levels of ALP and RUNX2 staining in control, force with or without Fludarabine treated MSCs. (E) The chondrogenic differentiation of MSCs in control, force with or without fludarabine treated groups were assessed by Alcian blue staining. (F) The protein expression levels of chondrogenic markers SOX9 and ACAN in control, force with or without fludarabine treated MSCs. Scale bar: 100 μm; data are presented as the mean ± SEM, ∗P < 0.05; ∗∗P < 0.01; ∗∗∗P < 0.005.

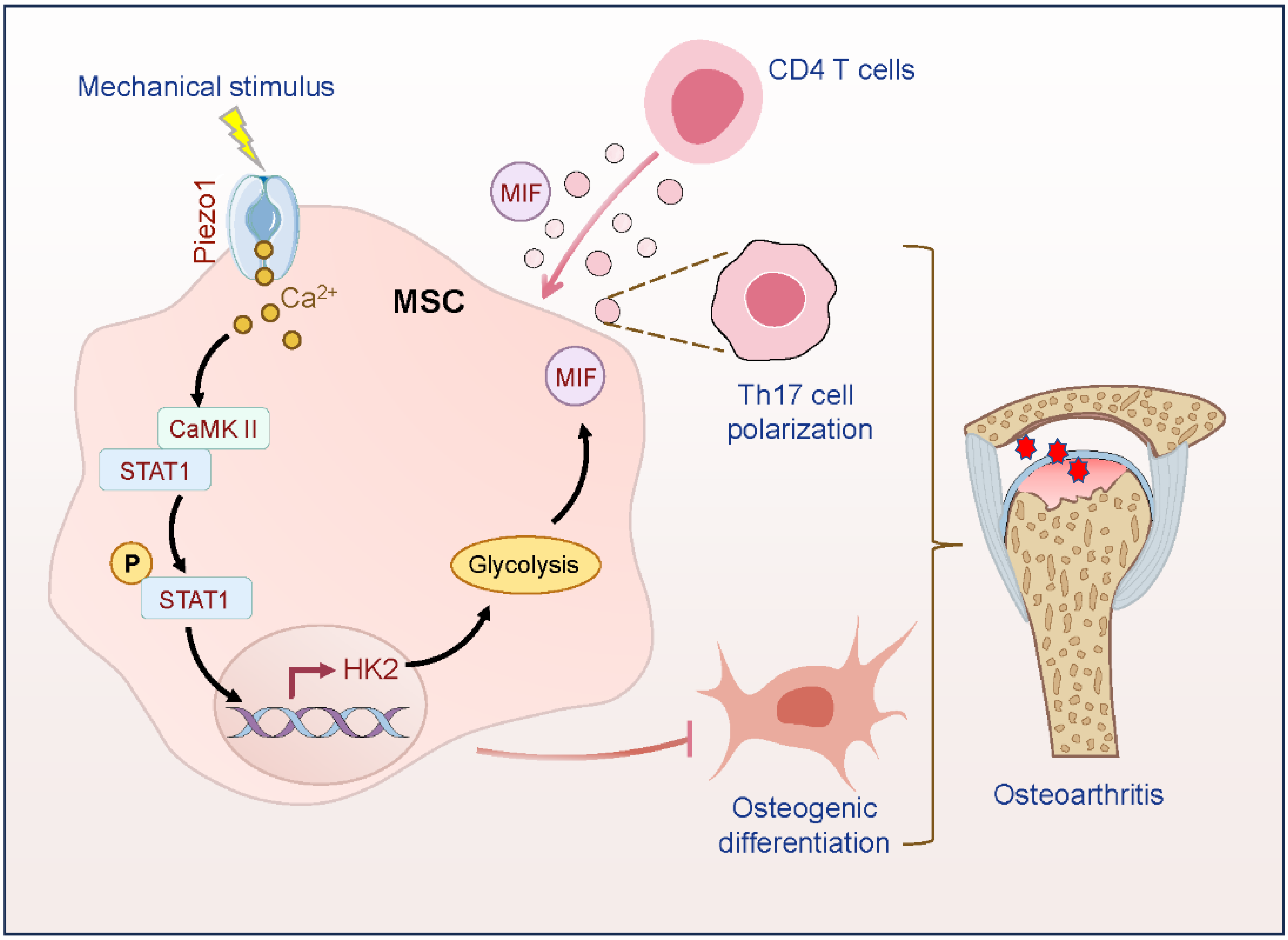

Supplemental Figure 7. The schema shows that mechanical sensing protein PIEZO1 in MSCs controls osteoarthritis *via* glycolysis mediated MSCs and Th17 cells crosstalk in a MIF dependent manner.

Captions for Supplementary Tables S1 to S6

Supplemental Table S1: The different genes list between Control and Force groups.

Supplemental Table S2: The TMJOA samples used in this study.

Supplemental Table S3: The primers used for qPCR.
