# Supplementary material for: Mechanical sensing protein PIEZO1 controls osteoarthritis via glycolysis mediated mesenchymal stem cells-Th17 cells crosstalk
Source: Cell Death Dis. 2025 Apr 1;16(1):231. doi: 10.1038/s41419-025-07577-1 (PMC11961634; doi:10.1038/s41419-025-07577-1)
Supplement: Supplementary file 2 — Supplemantal Material-Uncropped western blot images [file 41419_2025_7577_MOESM2_ESM.pdf]

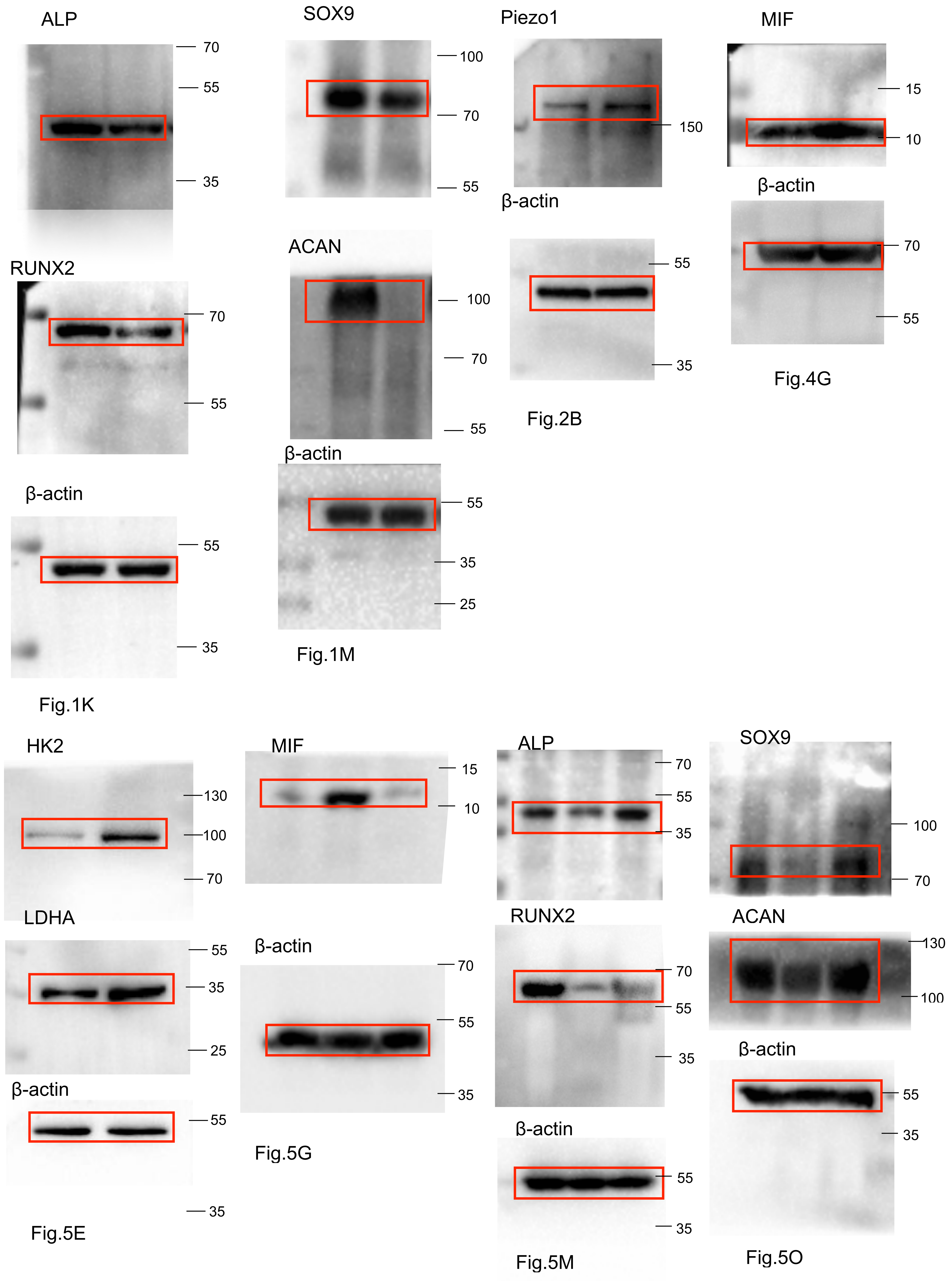

Supplementary Figure. Uncropped western blot images for Figure 1- 5

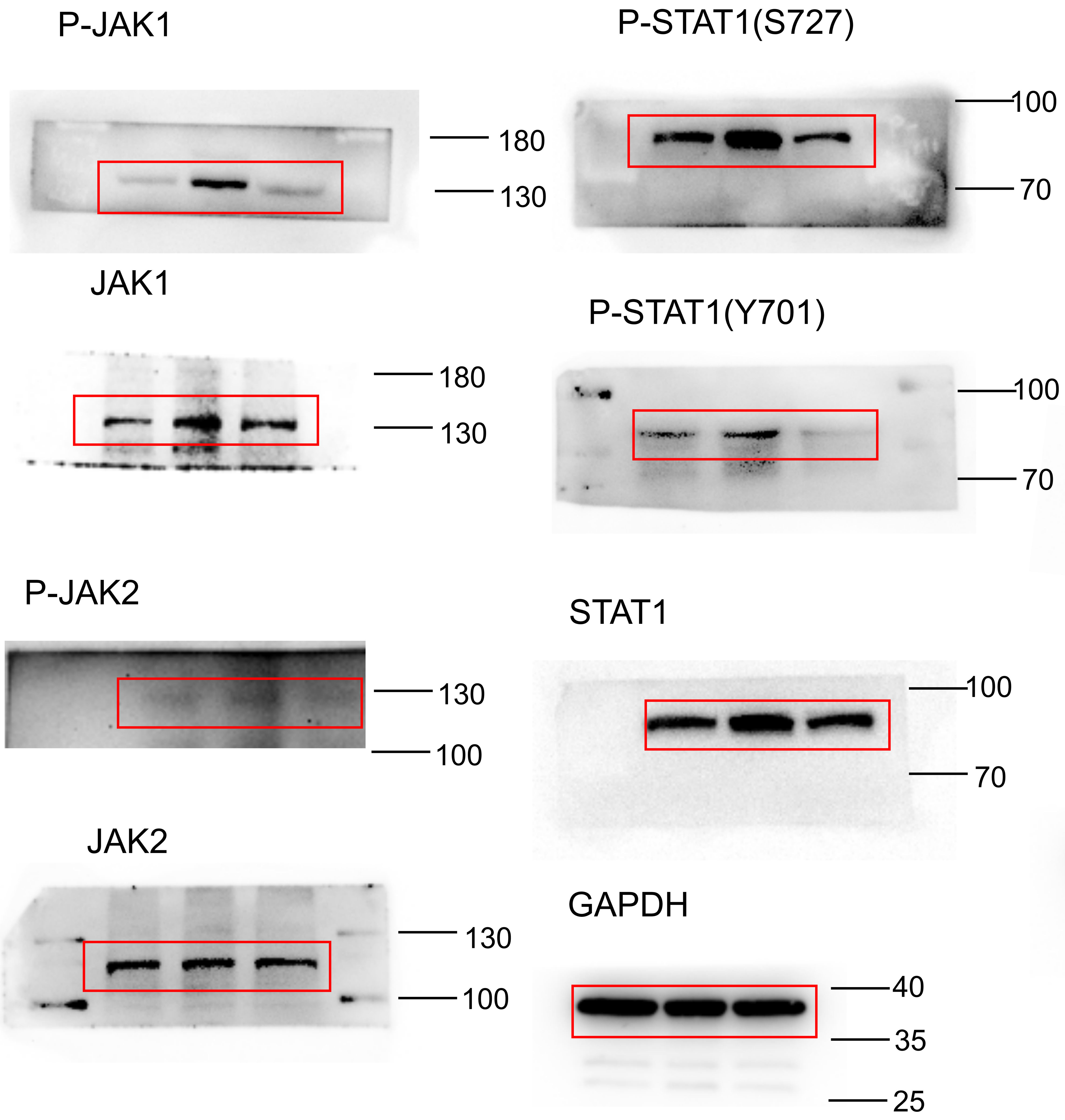

Fig.6A

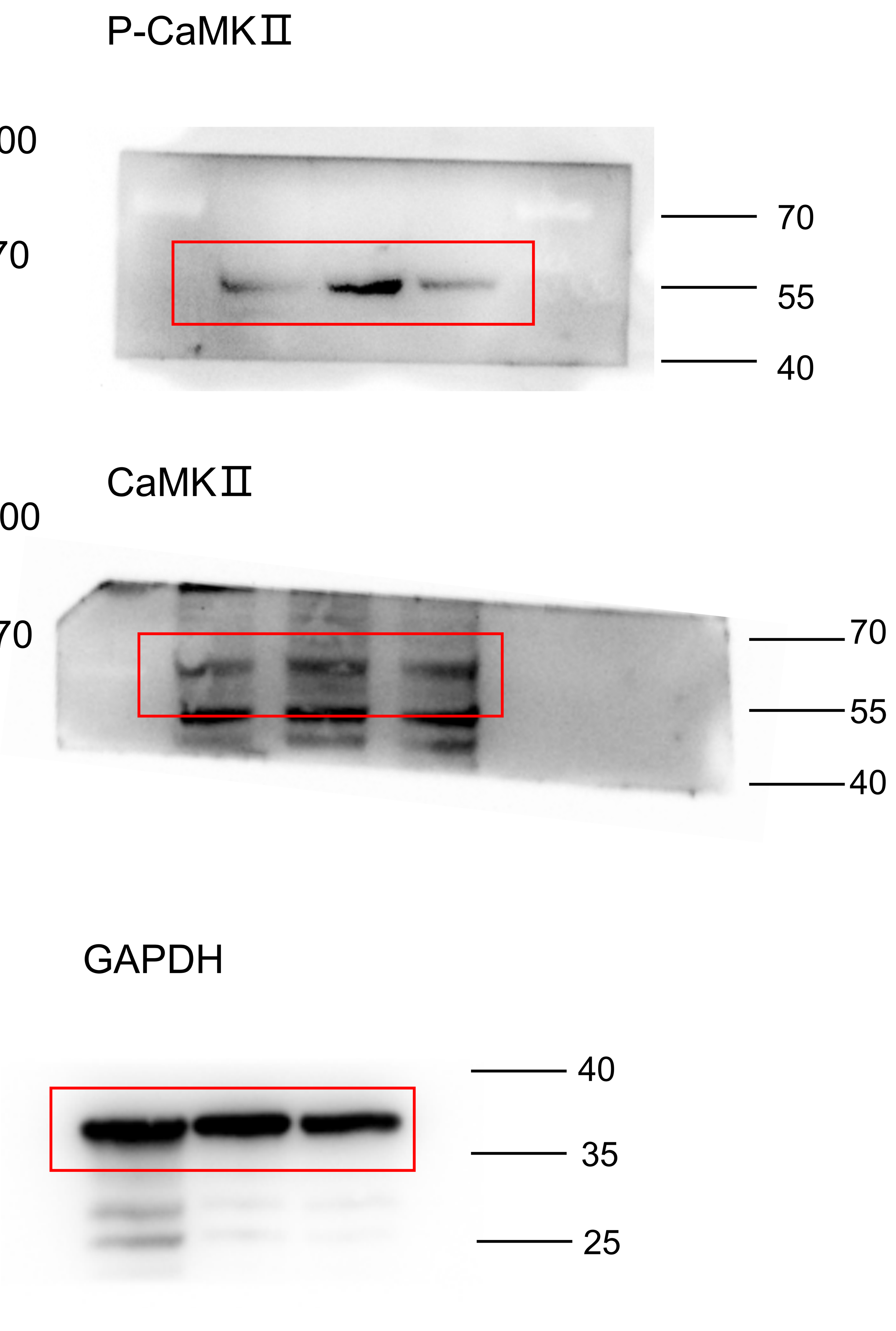

Fig.6E

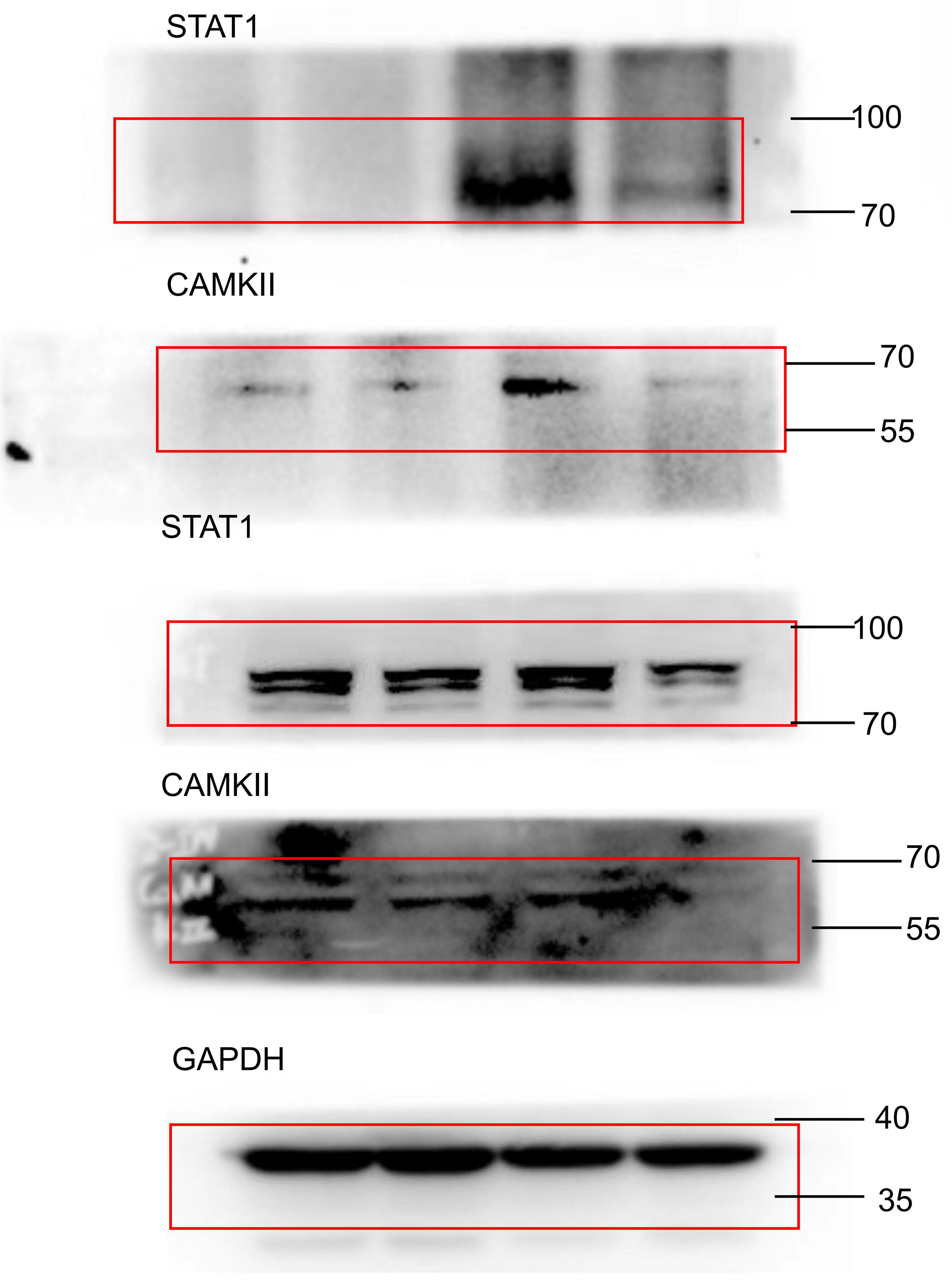

Fig.6F

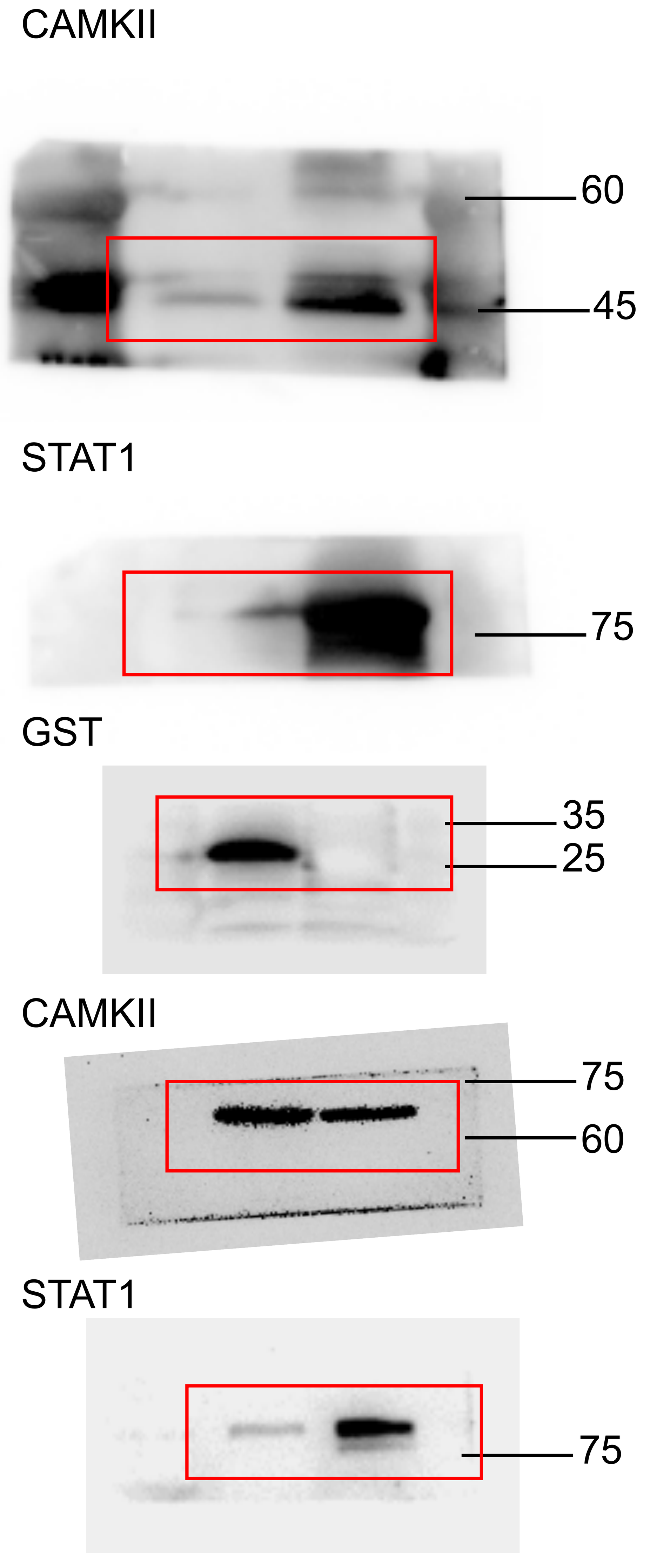

Fig.6G

Supplementary Figure. Uncropped western blot images for Figure 6A-6G

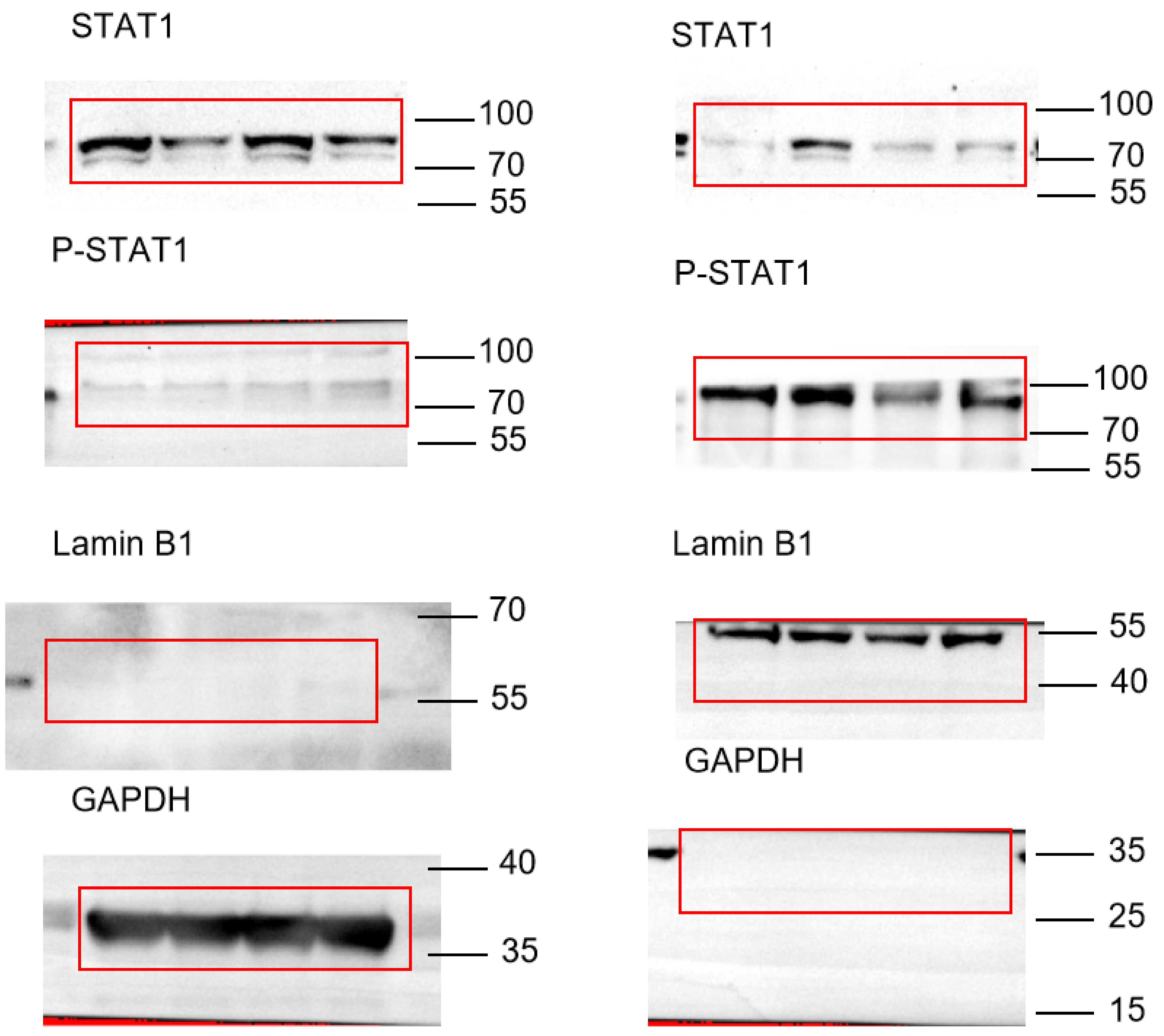

Fig.6H

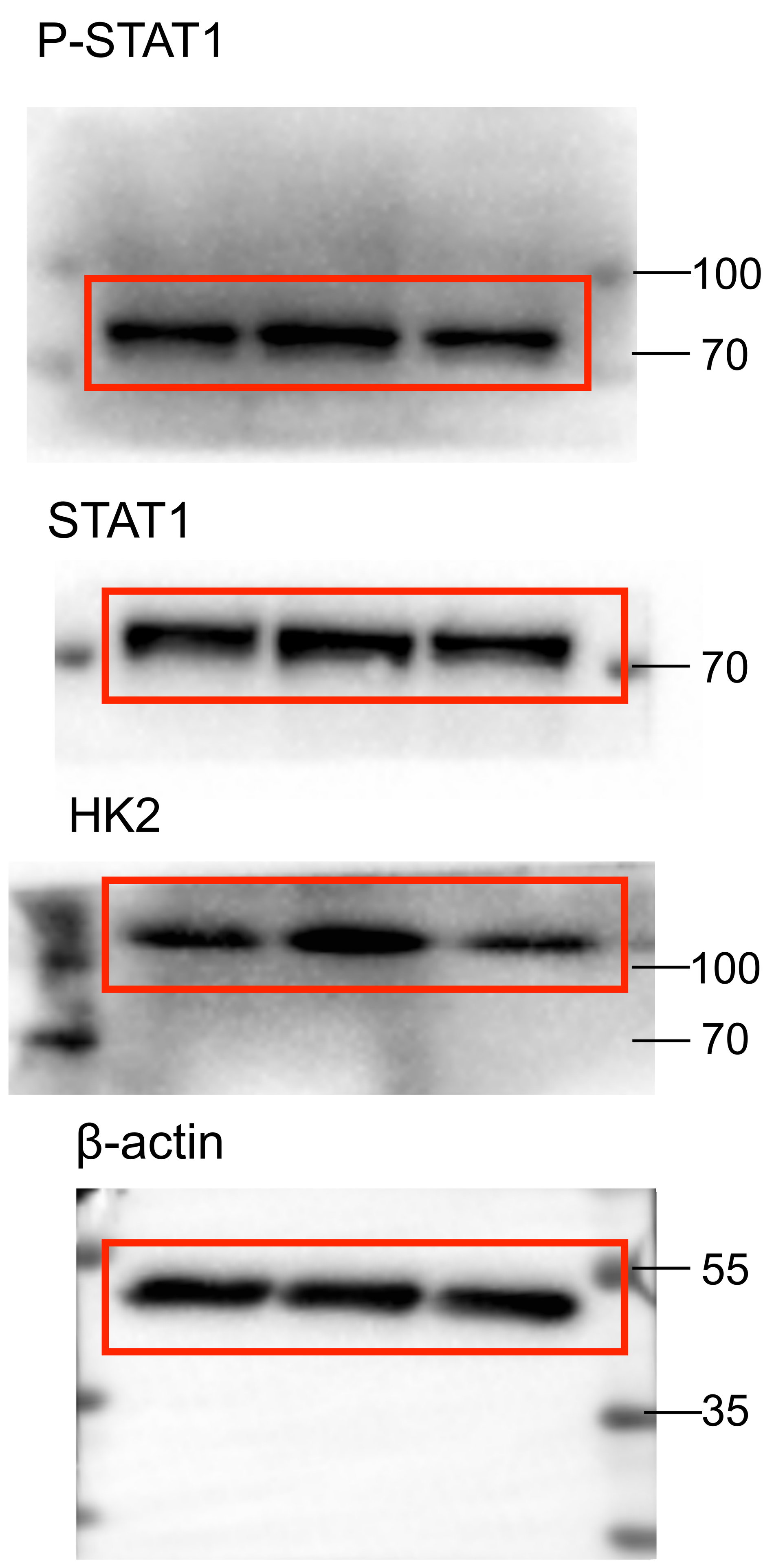

Fig.6I

Supplementary Figure. Uncropped western blot images for Figure 6H-6I

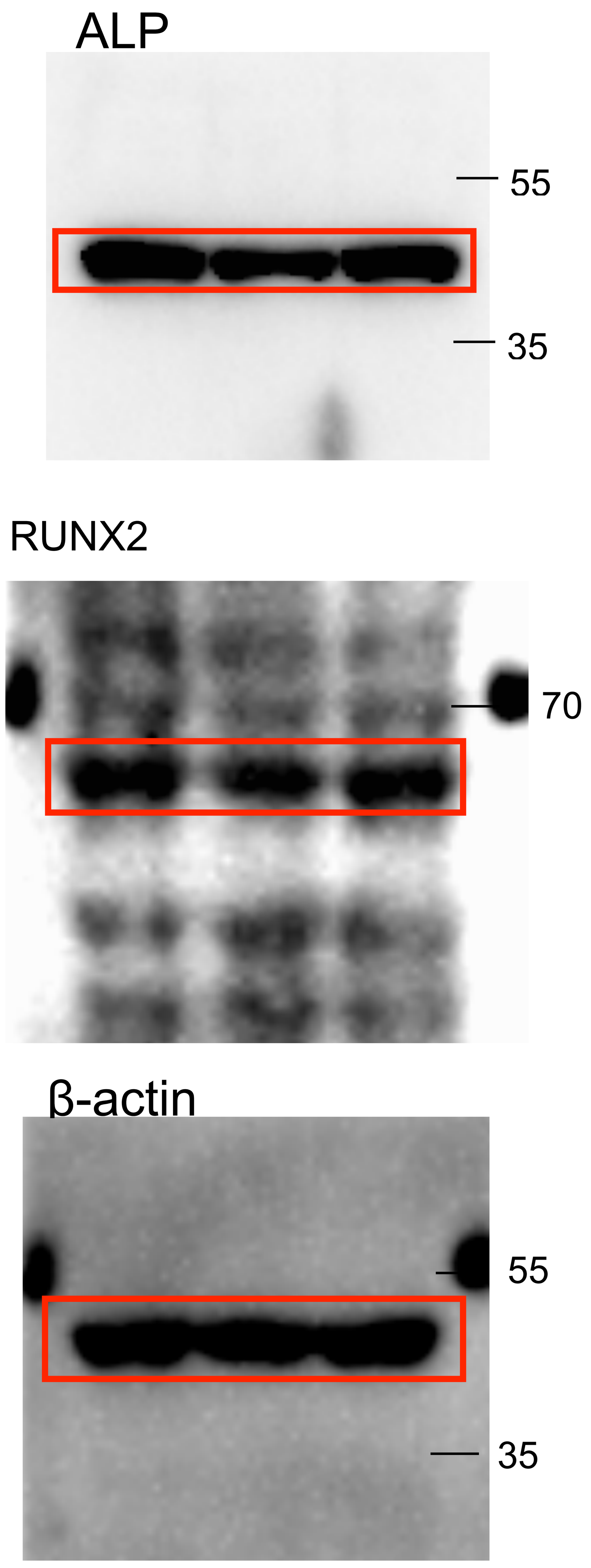

Fig.S2C

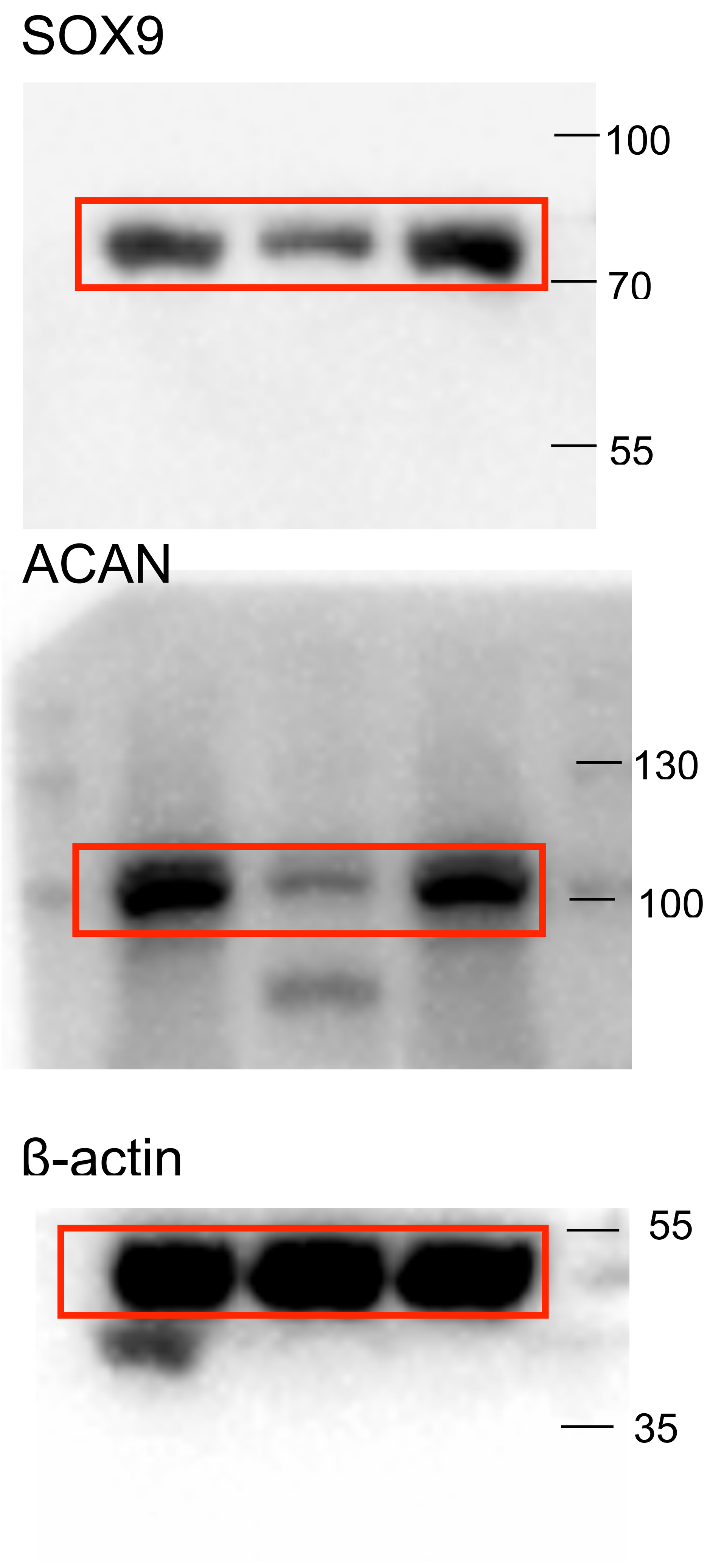

Fig.S2E

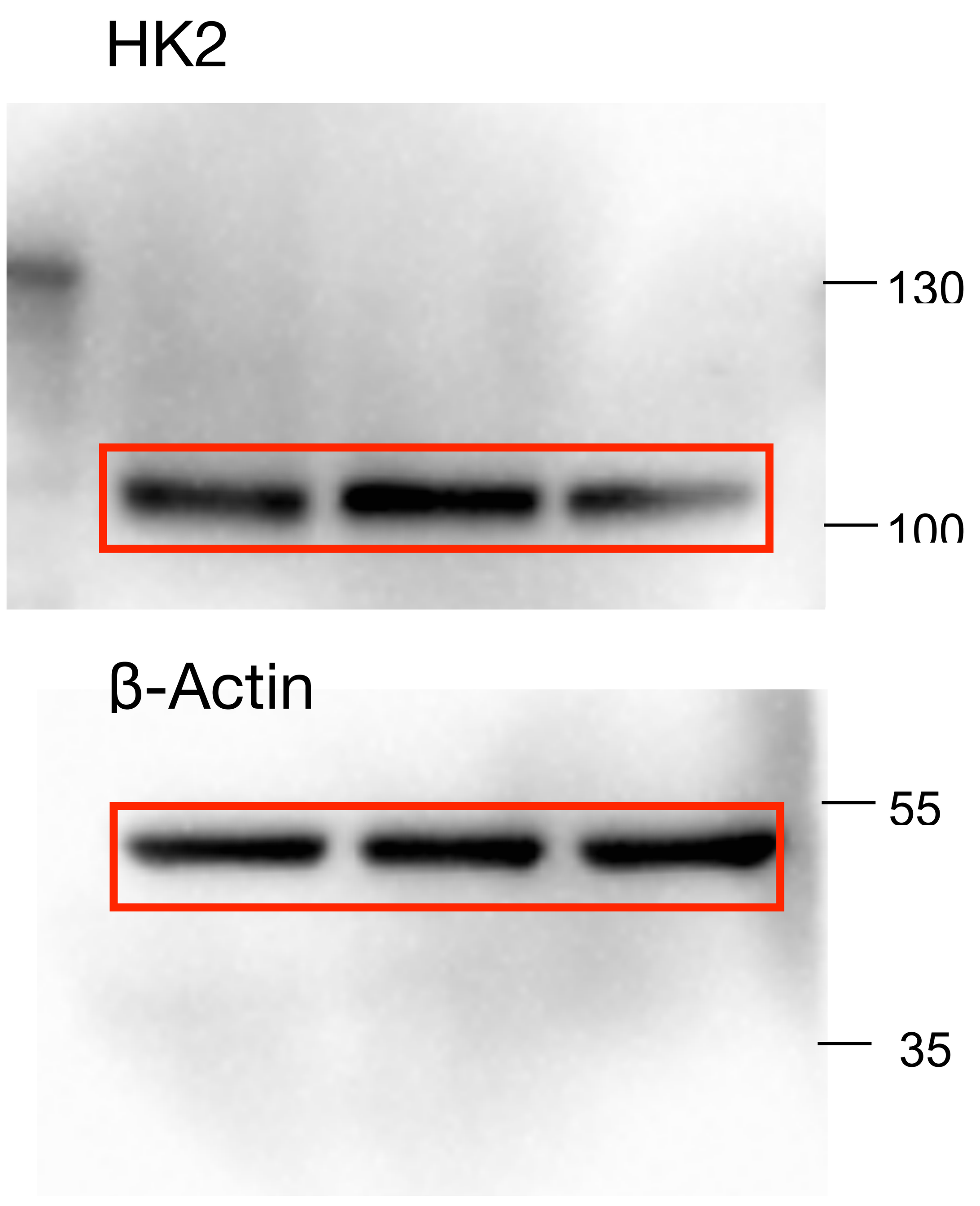

Fig.S5C

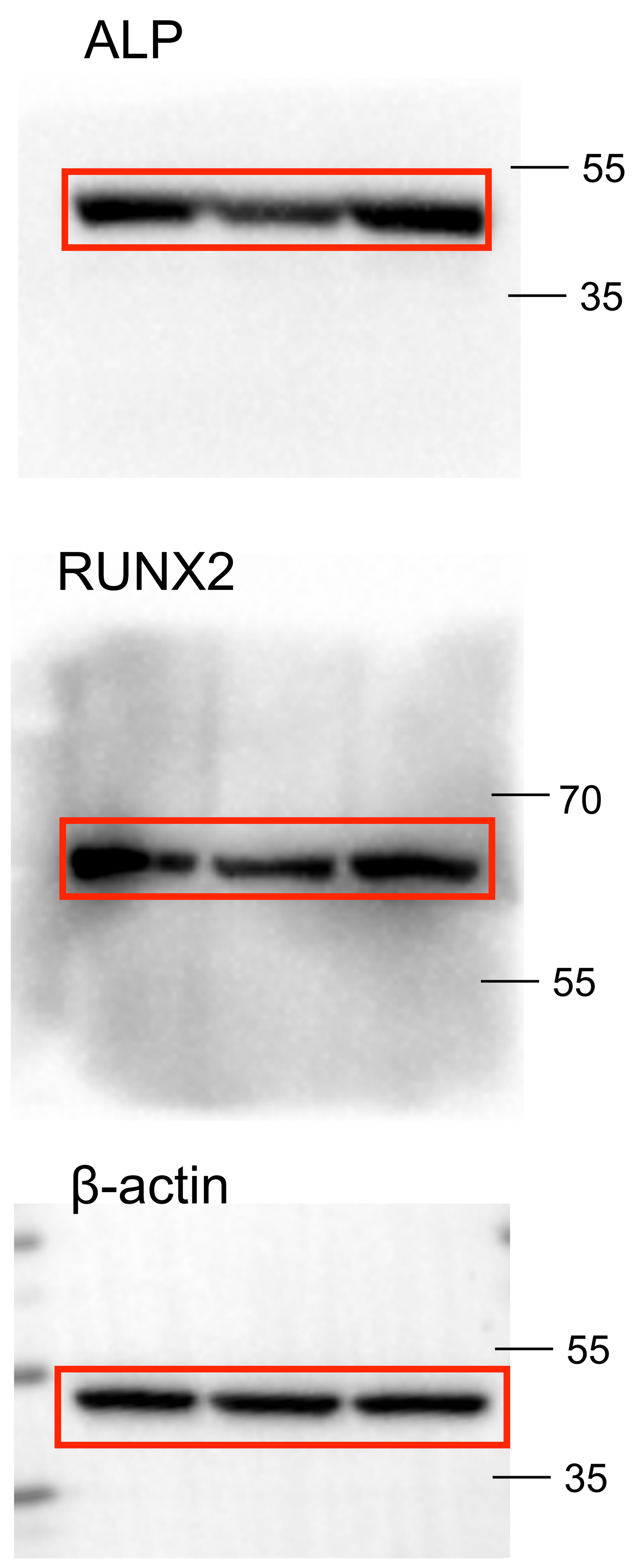

Fig.S6D

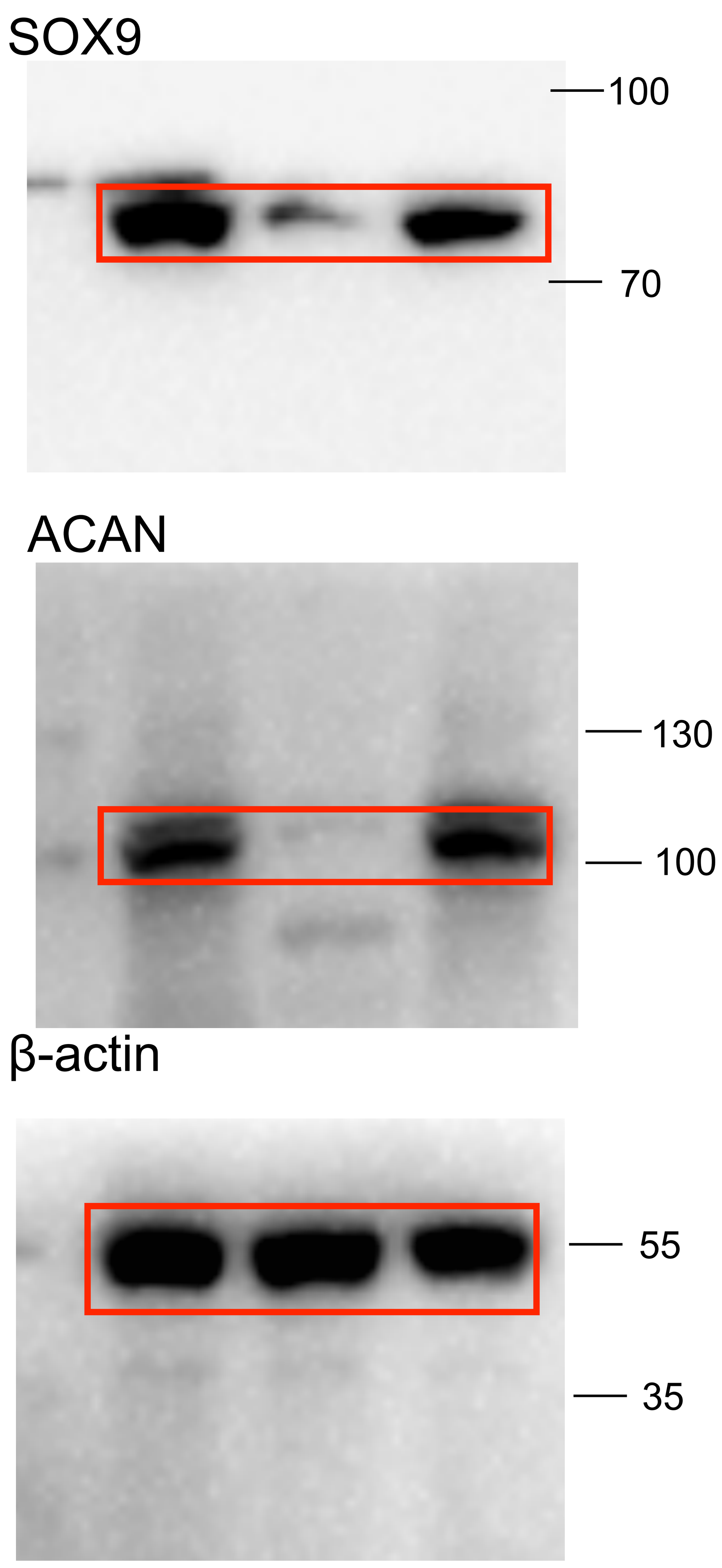

Fig.S6F

Supplementary Figure. Uncropped western blot images for upplementary Figure
